# A strategic mindset predicts and promotes effective learning and academic performance

**DOI:** 10.1038/s41539-025-00367-6

**Published:** 2025-10-31

**Authors:** Patricia Chen, Qiao Kang Teo, Daniel X. Y. Foo, Yifan Jiang, Lining Sun, Xiang Ling Ong, Delphinna Neo, Don J. H. Pereira, Bernard Tan, Khai Qing Chua, Joyce X. F. Gan, Desmond C. Ong

**Affiliations:** 1 The University of Texas at Austin, Austin, TX USA; 2 National University of Singapore, Singapore, Singapore

**Keywords:** Education, Psychology

## Abstract

Using effective learning methods is central to self-regulated learning and contributes to academic achievement. We examined one psychological factor—a strategic mindset—that predicts differences in effective strategy use, and intervened on it. A strategic mindset is an orientation toward asking oneself questions that elicit the access and use of task-appropriate methods, especially in moments of difficulty or unproductivity. To investigate the implications of a strategic mindset on real-world academic outcomes, we conducted four studies with 7475 adolescent and adult students from over 30 Singapore schools. In correlational surveys, a strategic mindset predicted greater use of effective learning strategies, and in turn, higher exam performance. In a field experiment with 1070 students, a strategic mindset intervention increased students’ reported use of effective learning strategies, and in turn, exam performance, among more academically prepared students and in conducive peer environments. This research advances self-regulated learning theory in a practically impactful way.

## Introduction

Self-regulated learners take responsibility for, and control of, their learning processes^1–3^. A central part of self-regulated learning involves reflecting on, using, and adapting learning methods that may be appropriate and effective for the task at hand^1,4–6^. These processes contribute to effective learning and academic performance^7–11^. Based on what they know about themselves and what is appropriate for the task, self-regulated learners access and apply various learning methods^1,12^. These methods may include metacognitive strategies (e.g., planning, self-monitoring, and self-reflection) and cognitive learning strategies (e.g., summarizing facts in one’s own words or connecting information to prior knowledge).

Decades of research in educational and cognitive psychology, especially that on self-regulated learning, has identified, measured, and intervened on strategies as specific skills to be taught and learned. This body of work has often focused on describing and measuring differences in the use of cognitive, motivational, and behavioral strategies between more versus less self-regulated learners^2,4,8,9,13–15^. For example, three of the most widely used instruments of learning strategies measure learning strategy knowledge and use, with some variation in their approaches. The Learning and Study Strategies Inventory (LASSI) measures students’ awareness and use of learning strategies during learning and testing, such as being able to identify key ideas during lessons (under the “Skill” subscale) and self-testing (under the “Self-Regulation” subscale)^4^. The Motivated Strategies for Learning Questionnaire (MSLQ) separates students’ use of cognitive (e.g., elaboration, organization), metacognitive (e.g., planning, monitoring), and resource management strategies (e.g., help seeking, time management)^8,9^. The Metacognitive Awareness Inventory (MAI) measures students’ knowledge of metacognitive strategies (such as when and how to implement each strategy) and their application of specific metacognitive strategies (e.g., monitoring, debugging)^14^.

Many interventions have attempted to make students aware of effective learning strategies and to train them in how to implement specific strategies (e.g., refs. ^16–19^). However, these methods are often manpower or resource intensive and difficult to scale (for exceptions, see refs. ^20,21^). Moreover, instructional approaches may not always adequately motivate students to spontaneously use effective strategies on their own^22^. Even when students are aware of effective strategies, many of them still do not apply these strategies when needed^22^.

To complement the aforementioned skills-focused approach, we test one psychological process that predicts effective strategy generation and use, and which could pave the way for scalable interventions to support students’ self-regulation: a strategic mindset. A strategic mindset is defined as an orientation toward frequently asking oneself questions that elicit the access and use of task-appropriate methods, such as “What can I try to be better at this?”, “How else can I do this?”, or “Is there a way to do this even better?”, especially when faced with difficulty or unproductivity^23,24^. Asking these questions prompts the assessment of current approaches, consideration of other, potentially better methods, and application of task-appropriate methods that may be effective for the task at hand.

A strategic mindset is distinct from a growth mindset of intelligence, and empirically predicts effective self-regulated learning over and beyond a growth mindset. A growth mindset of intelligence is the specific belief that intelligence is malleable, as opposed to fixed, innate, and unchangeable^25^. In contrast, a strategic mindset is not a belief about the malleability of personal attributes, but is instead a general tendency toward self-prompting strategy use^23^. In previous research (Study 2 from ref. ^23^), the two mindsets were weakly, but not significantly, correlated, and they emerged as separate factors in factor analyses. Additionally, a strategic mindset uniquely and significantly predicted effective learning strategy use above and beyond a growth mindset of intelligence.

A strategic mindset is related to metacognition—however, metacognitive processes encompass a much broader range of abilities and skills, whereas a strategic mindset is specifically the tendency to self-prompt strategy use, especially in moments of difficulty or unproductivity. Metacognition is, broadly speaking, the “ability to reflect upon, understand, and control one’s learning,” p. 460,^14^. As mentioned earlier, applying metacognition includes possessing metacognitive knowledge (of the self, of various strategies, and of when to apply which strategies) and implementing specific strategies (such as monitoring and debugging)^14^.

As we know from prior research, simply having awareness of effective strategies does not necessarily translate into practicing these strategies when needed. Learners can be aware of effective strategies, and yet not use these strategies at appropriate times^22^. For instance, in the face of difficulty or failure, many often fall into helpless responses, rather than reflect on their approaches and consider what they might do differently^26^. However, people with a strategic mindset make use of difficulty and unproductivity as cues to trigger metacognitive questioning. When they encounter such cues, they ask themselves strategy-eliciting questions, which prompt them to generate or seek out potentially better strategies. One of the contributions of our work on a strategic mindset is to specifically isolate and test the value of self-prompting strategy use. Especially in moments of difficulty or unproductivity, perhaps asking oneself questions such as “What can I try to be better at this?” or “Is there a way to do this even better” may facilitate the consideration and use of potentially better strategies.

Empirically, prior factor analyses found that strategic mindset scale items loaded onto a separate factor from items that measured people’s use of specific metacognitive strategies (e.g., planning, monitoring; adapted from the MSLQ), which loaded onto their own “metacognition” factor; importantly, none of the items cross-loaded highly onto the other factor (Studies 1 & 2 from ref. ^23^). Thus, a strategic mindset seems to be conceptually and empirically separable from general metacognitive strategy use.

Other studies indicate that a strategic mindset can predict and increase people’s use of metacognitive strategies. In a survey on American college students, a strategic mindset was associated with greater self-reported metacognition, and in turn, higher cumulative grade point averages (GPA, [ref. ^23^, Study 1]). Another study with entrepreneurs showed that while entrepreneurs who were more frugal with resources tended to engage in less action to benefit their venture, those with a strategic mindset showed the opposite pattern, where frugality actually predicted more action^27^—highlighting a link from a strategic mindset to resource regulation in a non-educational domain. In a randomized, controlled lab experiment, American adults and college students were asked to read a brief, online article on the value and practice of a strategic mindset, and then to summarize the main takeaways of that article as though they were sharing it with others on social media. The article conveyed that a strategic mindset is one key to success in life, described a strategic mindset, and emphasized the benefits of a strategic mindset. Notably, this strategic mindset article did not include any training in the use of specific metacognitive strategies. Participants who were randomly assigned to read and summarize the strategic mindset article (without any specific strategy training) later reported greater use of task-appropriate metacognitive strategies when performing a difficult, unfamiliar task, compared to participants in a control group who had not received any strategic mindset messaging (ref. ^23^, Study 3).

Additionally, experiments conducted with preschool children taught children a strategic mindset through stories and measured their use of self-regulatory strategies. Children were read a storybook featuring a character who had to wait for something they wanted across three different scenarios. Children who were randomly assigned to a strategic mindset condition learned that the character coped with waiting by singing a strategic mindset mantra: “What can I try? What can I try to be better at this?” Children were encouraged to sing this mantra alongside the character while reading each scenario. Despite receiving no strategy instruction, children who were taught this strategic mindset mantra subsequently demonstrated greater use of effective self-regulatory strategies on two separate delay-of-gratification tasks, relative to those who read comparable stories of waiting without the strategic mindset content^28^. Evidently, experimentally manipulating a strategic mindset translates into measurable changes in strategy use—at least on laboratory tasks. Could this translate to real-world learning outcomes?

Although a strategic mindset has important implications for learning, problem-solving, and goal progress in general^23,27–29^, limited empirical research has examined its associations with, and causal implications for, real-world academic outcomes. Our goals in this paper were two-fold: One, we aimed to examine to what extent a strategic mindset can predict, and causally facilitate, greater use of effective learning strategies, and in turn, exam performance. Two, we sought to develop and test a cost-effective, scalable way of intervening on a strategic mindset.

We hypothesized that students’ strategic mindset would prospectively and positively predict students’ greater use of effective learning strategies, and by doing so, support better academic performance. Frequently asking oneself strategy-eliciting questions (such as “What can I do to make myself better at this?”) can motivate students to access and apply more effective strategies during learning. As a great deal of scientific literature has shown, the more students apply effective strategies when learning, the better they tend to perform academically^7–11^. Thus, we expected the relation between students’ strategic mindset and academic performance to be mediated by their use of effective learning strategies—because it is through such effective learning behaviors that students’ strategic mindset questions might actually translate into better performance.

We tested this hypothesized psychological process among 7475 adolescent and adult students in Singapore. Singapore students speak English as their official language and they have a rigorous, challenging curriculum; Singapore students consistently score at the top of the Programme for International Student Assessment (PISA) rankings, attesting to the strong support for quality education and effective student learning approaches that make their education system worthy of understanding and modeling. Conducting these studies on English-speaking, non-Western students helps us test whether prior research on a strategic mindset (and the psychological process by which it is theorized to work) generalizes beyond American students and adults.

Arguably, every learner will experience difficulty or unproductivity at some point or another in life. Being able to figure out what methods are appropriate and effective to navigate learning is important, especially in these moments of difficulty and unproductivity^30,31^. Because we wanted to understand whether a strategic mindset plays an important role in effective learning, both under normal learning circumstances and during difficult, changing circumstances, our studies spanned the COVID-19 pandemic period and the post-pandemic era. Our theory was that a strategic mindset would be useful under normal learning circumstances, and also useful in the face of learning difficulties and changes. Therefore, testing the implications of a strategic mindset under both of these kinds of circumstances allowed us to better understand its contextual generalizability.

We first ran two correlational surveys of students to establish the relations among a strategic mindset, learning strategy use, and exam performance; after which, we added two randomized, controlled experiments as causal tests of the psychological process. To understand the real-world implications of a strategic mindset for learning and performance, Study 1 surveyed a nation-wide sample of 5185 Primary 6 to Secondary 4 (ages 12–16; International Standard Classification of Education (ISCED) Level 2–3 and the last year of Level 1^32^ equivalent to U.S. grades 6 to 10) students in Singapore, as they navigated their learning amidst the educational changes and difficulties brought about by the COVID-19 pandemic. Study 2 then replicated our findings in an older student sample—among 1037 college-level undergraduates (ISCED Level 6)—who navigated a mix of online, in-person, and hybrid classes during the pandemic.

Next, we developed an online, scalable intervention that could be self-administered by students, and tested its efficacy in two experiments: Experiment 3 was an initial test of the online intervention effects on 183 college students’ intentions to apply effective strategies to learn. Experiment 4 involved a larger, randomized, controlled field trial among 1070 Secondary 2 to 4 (ages 14–16; ISCED Level 2–3; equivalent to U.S. grades 8 to 10) students. This field experiment tested both the efficacy of the intervention and heterogeneity in its effects on students’ reported strategy use when studying for their final exams, and consequently, on their final exam performance.

## Results

### Study 1: nation-wide survey of adolescents

We tested the role of a strategic mindset in students’ use of effective learning strategies and academic achievement amidst the changes and difficulties wrought by the COVID-19 pandemic. We conducted a nation-wide survey of adolescents in Singapore, assessing the relations among their strategic mindset at the beginning of the 2020 academic year, their reported use of effective learning strategies in the middle of the academic year (amidst the challenging, pandemic-necessitated learning transitions from in-person to online and back to in-person lessons), and their final exam performance collected at the end of the school year. We predicted that students’ strategic mindset would prospectively and positively predict their use of learning strategies that are scientifically proven to be effective for mastery (e.g., relating new knowledge to their existing knowledge, identifying the important content to study), and consequently, that these students would perform better on their year-end, final exams.

We surveyed 5185 late primary through secondary students (across 5 grades: approximately 11–12 through 15–16 years old) from 270 classes across 29 Singapore public schools. A detailed demographic breakdown is provided in Supplementary Table 1 in the Supplementary Information (SI).

At the beginning of the academic year (February to March), we assessed students’ strategic mindset using a 5-point response scale (*1=Never*, *5=Most of the time*). As mentioned earlier, people with a strategic mindset use moments of difficulty or unproductivity as cues to ask themselves strategy-eliciting questions. Therefore, the strategic mindset scale items take the form of how often students ask themselves such strategy-eliciting questions, especially when they encounter difficulty or unproductivity. Examples of strategic mindset scale items include: “When something is very hard for you, how often do you ask yourself: ‘What can I do to make myself better at this?’” and “Whenever you feel like you are not making progress, how often do you ask yourself: ‘Is there a different way I can do this?’”

In the middle of the academic year (July–August), students experienced a month-long learning transition from in-person classes to fully online learning, and then back to in-person classes. Shortly after they returned to in-person classes, we asked students to report how much they had been using various effective learning strategies during learning. We used the well-established Learning and Study Strategies Inventory (LASSI) 3^rd^ edition Skill subscale^4^, which included items such as: “I summarize what I’m studying in my own words,” “I try to connect what I am studying to what I already know,” and “When my teacher is teaching a lesson in class, I am able to pick out the important information.” In the “Methods” section, we describe each of our scale measures in further detail, along with their scale reliabilities, for all studies. At the end of the school year, with parental consent and students’ assent, we obtained students’ final exam performance results.

To analyze the effect of a strategic mindset on learning strategies and on final exam performance, we used mixed-effects models using the *lme4* package in R, including random effects of level and school, and of class nested within level and school. This is because students are in only one class, classes are nested within grades and within school, and grade-levels and schools are crossed (see Supplementary Note 1 for further details about the analytical approach and explanation). For mediation analyses, we used the *Process* macro model 4^33^ with 1000 bootstrap resamples.

As hypothesized, students’ strategic mindset prospectively and positively predicted the degree to which they reported using effective learning strategies amidst the challenging mid-year learning transitions (mixed effects model regression coefficient *b* = 4.07, [3.73, 4.41], *t* = 23.45, *p* < 0.001); and in turn, students who used effective learning strategies to a greater degree performed better on their final exams (mixed effects model regression coefficient *b* = 0.21 [0.19, 0.24], *t* = 16.83, *p* < 0.001).

**Fig. 1:**
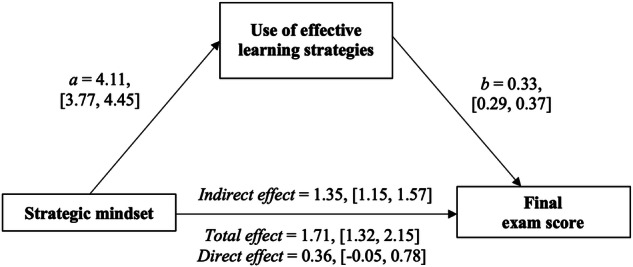
Mediation model showing how students’ strategic mindset predicts end-of-year academic performance through use of effective learning strategies. The figure represents results from a simple mediation model. The model shows the relation between students’ strategic mindset at the beginning of the academic year and their year-end, final exam performance, mediated by their reported use of effective learning strategies in their classes. Unstandardized coefficients (represented by the small letters) and their 95% confidence intervals (in square brackets) were bootstrapped 1000 times.

We found the predicted indirect effect of a strategic mindset on final exam performance, mediated by greater use of effective learning strategies (indirect effect estimated with 1000 bootstrap resamples = 1.35, [1.15, 1.57]; Fig. 1). When accounting for the indirect effect, the direct effect of a strategic mindset on final exam performance was diminished. This is consistent with what we might expect when students simply ask themselves, “What can I do to make myself better at this?” but do not actually apply task-appropriate, effective strategies.

As a robustness test, the observed indirect effect was still significant when controlling for students’ prior year final exam performance (indirect effect with prior performance covariate = 0.28, [0.19, 0.37])—even though their prior performance was highly correlated with their current final exam performance at *r* = 0.85, on average across cohorts. Our results were also robust when controlling for students’ growth mindset of intelligence, which is a well-known mindset construct that reliably predicts academic achievement, as described earlier (indirect effect with growth mindset as a covariate = 1.16, [0.97, 1.36]).

Importantly, this mediated psychological process model was robust across every grade level (Table 1)—including the crucial Primary 6 and Secondary 4 years, when students in Singapore sit for high-stakes, standardized national exams. These national exams are the Primary School Leaving Examination (PSLE) and the GCE O-Level and N-Level Examinations taken at the end of Secondary School, respectively (the “Methods” section provides further details about these exams and how they are scored). Unlike school-specific exams, the PSLE and GCE Examinations are administered in a coordinated, consistent manner at the same time across schools nation-wide to all students within the same cohort, which makes individual students’ performance scores comparable across classes and schools at a national level. These national exams are important for students’ long-term educational trajectories. That is, performance on these exams determines whether they qualify to proceed to the next educational level, the schools that they are eligible to enroll in, and the subjects that they can study.

**Table 1:** Mediated effect of a strategic mindset on final exam and national exam performance (with use of effective learning strategies as the mediator) aggregated across schools, broken down by grade level

Performance outcome by grade level	Sample size, *N*	Indirect effect, *ab*	95% CI
Primary 6 Year-End Exam	852	2.38	[1.81, 3.00]
Primary 6 PSLE National Exam	873	1.97	[1.53, 2.45]
Secondary 1 Year-End Exam	668	0.93	[0.60, 1.31]
Secondary 2 Year-End Exam	603	0.93	[0.60, 1.30]
Secondary 3 Year-End Exam	713	0.57	[0.25, 0.94]
Secondary 4 Year-End Exam	542	0.96	[0.57, 1.43]
Secondary 4 GCE O-Level National Exam	371	1.06	[0.32, 1.94]
Secondary 4 GCE N-Level National Exam	99	1.37	[0.41, 2.59]

This table shows the indirect effect *ab* (with 95% confidence interval) estimates of a strategic mindset on examination performance at each grade level. The mediator in the model is students’ reported use of effective learning strategies. We present the school-based final exam scores in each row (out of a total of 100 percentage points), along with the standardized national exam scores for the PSLE (taken at the Primary 6 level) and the GCE Examinations (taken at the Secondary 4 level, either at the O-Level or the N-Level, depending on the student’s track). Primary 6 to Secondary 4 corresponds to U.S. Grades 6 to 10. These indirect effects were calculated based off percentage-point equivalents of the raw exam scores, which were transformed for interpretability in this table. Please refer to the Supplementary Note 1 for details of how we transformed the raw national exam scores to percentage-point equivalents. All the indirect effects for each level are statistically significant, which can be inferred from the 95% confidence intervals. Sample size *N* gives the sample size for that analysis after pairwise deletions for missing data (e.g., if we were unable to get their year-end or national exam scores).

To get a sense of the practical effect size, Supplementary Table 2 provides a breakdown of the raw performance scores by education level, organized by type of exam. A 1-point increase on the 5-point strategic mindset scale, acting through students’ increased use of effective learning strategies, corresponded to scoring an average of 5.9 raw points higher on students’ national Primary School Leaving Examinations at the Primary 6 level. Likewise, a 1-point difference on the strategic mindset scale corresponded to scoring about 0.5 points lower on the Secondary 4 O- and N-Level Examinations, where lower scores reflect higher academic achievement.

We also observed a significant, positive total effect of a strategic mindset on exam performance. Aggregating across levels and schools, students who scored higher on a strategic mindset at the beginning of the year performed better on their final exams (mixed effects model *b* = 1.03, [0.77, 1.29], *t* = 7.78, *p* < 0.001). A 1-point increase on the strategic mindset scale corresponded to scoring an average of 1.03 percentage points (i.e., out of 100) higher on the final exams.

To summarize our first study, we found the hypothesized relation between a strategic mindset, students’ reported use of effective strategies during learning, and their academic performance. This psychology seems to be robust across a range of educational levels, types of exams (both school-specific and standardized, high-stakes national exams), and even when controlling for the aforementioned covariates that tend to be relevant to academic achievement. Our results underscore the important, potentially generalizable effects of a strategic mindset on effective learning strategy use, and in turn, real-world performance outcomes.

### Study 2: college student replication study

To test the robustness and further generalizability of our findings to older students, we replicated our findings with 1037 final-year Singapore public university undergraduates (*M*_age_ = 23.9 years, SD_age_ = 1.2, range: 21–30 years. 59.4% females) in a pre-registered study. Participating students were recruited from various fields and Departments across the university (e.g., Business, Arts and Social Sciences, Engineering, Science, Design). The cross-sectional survey ran from March 2021 to mid-May 2021, when students were enrolled in online, in-person, and/or hybrid classes that were structured to accommodate the pandemic. These educational changes presented generally unfamiliar challenges to students, which provided an opportunity for us to study students’ self-regulated strategy use amidst such circumstances.

Participating university students completed measures of their strategic mindset using the strategic mindset scale for adults^23^ and reported how much they used various learning strategies known to be effective for learning (using the LASSI 3^rd^ edition Skill subscale for college students^4^; example item: “I try to find relationships between what I am learning and what I already know.”). With students’ consent and as our performance outcome of interest, we obtained from the university their 5-point end-of-semester GPAs (also known as their “Cumulative Average Points”) aggregated across all their classes for that academic semester.

Because this was a cross-sectional survey administered at a single time point, we used linear regression analyses to estimate the effect of a strategic mindset on students’ reported use of effective learning strategies, and in turn, their end-of-semester GPAs at the individual student level. Again, we observed that college students’ strategic mindset predicted their reported use of effective learning strategies in their classes (*b* = 5.56 [4.81, 6.31], *t* = 14.47, *p* < 0.001); and these students who reported using effective learning strategies to a greater degree attained higher GPAs at the end of that same semester (*b* = 0.01 [0.01, 0.01], *t* = 6.36, *p* < 0.001).

**Fig. 2:**
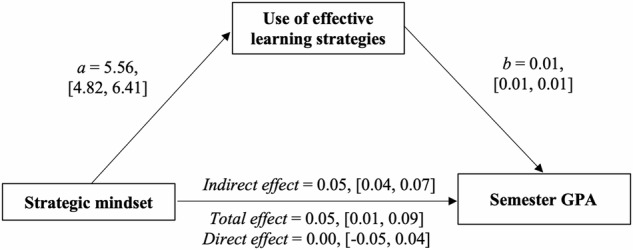
Mediation model showing how college students’ strategic mindset predicts semester GPA through use of effective learning strategies. The figure represents results from a simple mediation model. The model shows the relation between students’ strategic mindset and their 5-point GPA for that same semester, mediated by their reported use of effective learning strategies. Unstandardized coefficients (represented by the small letters) and their 95% confidence intervals (in square brackets) were bootstrapped 1000 times.

To test mediation, we used the lavaan package in R (Version 0.6-15^34^) with 1000 bootstrap resamples. We observed the predicted, significant indirect effect of a strategic mindset on semester GPA, mediated by students’ reported use of effective learning strategies during learning (indirect effect estimated with 1000 bootstrap resamples = 0.05 [0.04, 0.07]; Fig. 2). Like Study 1, the direct effect of strategic mindset on semester GPA was diminished when accounting for the indirect effect.

Our mediation results were the same when we log-transformed students’ semester GPA (which was negatively skewed; indirect effect = 0.01, [0.01, 0.02]), and also when we controlled for students’ prior academic achievement (specifically, their college admission scores; indirect effect with college admission scores as a covariate = 0.05, [0.03, 0.07]).

Overall, college students who had more of a strategic mindset also attained higher college GPAs that semester (total effect *b* = 0.05 [0.01, 0.09], *z* = 2.62, *p* = 0.009). For an idea of the practical effect size, scoring 2 points higher on the 5-point strategic mindset scale corresponded to scoring an average of 0.10 higher on their 5-point GPA. This practical effect, though small, replicated in both student samples and is associated with important, objective performance outcomes.

To summarize, our field Studies 1 and 2 show that students with more of a strategic mindset tend to apply effective learning strategies to a greater extent, and consequently, achieve better academic performance. We observed this psychology across adolescents and adults, even amidst learning changes and difficulties, and also on high-stakes, nationally standardized exams. Students with more of a strategic mindset were able to learn and to perform better than their peers who had entered college at comparable levels of prior achievement. Our findings underscore that a strategic mindset predicts learning strategy use in a way that matters for real-world learning and long-term educational outcomes of importance.

### Experiment 3: development and initial testing of an online intervention

In the next two experiments, we extended our correlational findings to randomized, controlled experiments (RCTs) that would allow us to test causality. Such RCTs are the gold standard for inferring causality, and they enable us to rule out alternative explanations (e.g., confounds, third variables) that may explain our correlational data^35–37^, such as a pre-existing repertoire of learning strategies or intelligence. Importantly, as part of our experimental testing, we developed online, scalable, cost-effective interventions to foster a strategic mindset that adolescents or university-level students could self-administer on their own.

Here, we developed a first strategic mindset intervention, which involved learning about the value of a strategic mindset and how to apply it. We shared with students persuasive anecdotes from the lives of highly successful people (e.g., famous athletes, businesspeople, scientists) who exemplified a strategic mindset, insights from scientific research about the importance of this mindset, and examples of how other students might apply and benefit from a strategic mindset. Students were asked to apply what they had learned in two ways: one, to write to a person close to them how that person might practice and benefit from using a strategic mindset; two, to write about how they themselves had previously applied, or were planning to apply in the future, a strategic mindset to achieve an important goal or to overcome challenges. These kinds of “saying-is-believing” exercises facilitate internalization and ownership of the intervention message, generating self-persuasion effects^38,39^. We present example screenshots of our intervention in Supplementary Note 2.

To establish the efficacy of our intervention approach before adapting the intervention for younger students and for in-classroom experiments, we first conducted a randomized, controlled experiment with 183 undergraduates (*M*_age_ = 21.5 years, SD = 2.3 years; 131 females, 51 males, 1 “other” gender; 164 Chinese, 11 Indian, 4 Malay, 4 “other” races) recruited from a large, public university in Singapore.

Participants were randomly assigned to either a strategic mindset condition or a control condition. Students in the strategic mindset condition received the strategic mindset intervention described earlier, whereas those in the control condition received similarly-structured messages about the value of, and how to, take breaks. The control condition was similarly structured, but did not include any strategic mindset messaging: students in the control condition also saw persuasive anecdotes from highly successful people, scientific insights, and other students’ testimonials. They also engaged in similar saying-is-believing writing exercises about the topic of taking breaks.

Finally, students answered survey questions about their attitudes toward adopting a strategic mindset, which was our manipulation check (example item: “Whenever something feels difficult, I should always ask myself: ‘What are things I can do to make myself better at this?’”; 7-point scale). Since research shows that intentions are an antecedent to behavior^40^, we wanted to know if self-administering the intervention would even have an effect on students’ intentions to practice a strategic mindset in the future. Students rated their future intentions to apply a strategic mindset (example item: “Whenever something feels difficult, I will ask myself what I can do to help myself get better.”; 7-point scale) as an additional outcome variable.

As our critical outcome of interest, students rated their intentions to use learning strategies that are effective for mastery over the next 2 weeks, using a prospectively-worded, abbreviated version of the LASSI Skill subscale described in Study 2 (example item: “Whenever I’m learning, I will try to find relationships between what I am learning and what I already know.”; 0–100% frequency scale). To test that our strategic mindset intervention specifically changes a strategic mindset and not a growth mindset of intelligence, we included a post-intervention growth mindset of intelligence measure (example item: “Your intelligence is something about you that you can’t change very much.”; 7-point scale).

Results showed that this initial test of the intervention was effective. Compared to the control group, students who were randomly assigned to receive the strategic mindset intervention reported stronger attitudes toward a strategic mindset (*M*_*SM*_ = 5.9 vs. *M*_*C*_ = 5.5, Welch’s *t* = 2.96, *p* = 0.003, Cohen’s *d* = 0.44), and greater intentions to apply it (*M*_*SM*_ = 5.6 vs. *M*_*C*_ = 5.3, Welch’s *t* = 2.55, *p* = 0.012, *d* = 0.37), compared to controls. Their self-reported attitudes and intentions were correlated at *r* = 0.44, *p* < 0.001. Importantly, students in the strategic mindset condition also reported greater intentions to use effective learning strategies over the subsequent 2 weeks of classes (*M*_*SM*_ = 23.2 vs. *M*_*C*_ = 21.9, Welch’s *t* = 2.00, *p* = 0.047, *d* = 0.30), compared to those in the control condition. The size of the effects on these 3 key outcomes were generally small (Cohen’s *d* ranged from 0.30 to 0.44), which is typical with brief, scalable social-psychological interventions and social-psychological effects in general^41,42^. There was no significant difference between conditions in students’ post-intervention growth mindset of intelligence beliefs (*M*_*SM*_ = 4.3 vs. *M*_*C*_ = 4.5, Welch’s *t* = 0.72, *p* = 0.470), indicating that the intervention specifically targets a strategic mindset. Overall, these findings gave us confidence to adapt the intervention for field testing with larger samples of students in classrooms.

### Experiment 4: randomized, controlled test of a self-administered strategic mindset intervention in secondary school classrooms

Next, we adapted and experimentally tested our online strategic mindset intervention in a randomized, controlled field experiment in Secondary school classrooms. Our goals were two-fold: (1) to test whether intervening on a strategic mindset would causally impact students’ learning strategy use, and in turn, final exam performance, and (2) to understand what kinds of students it might benefit and under what conditions. This was the first test of a strategic mindset intervention for Secondary school students. If the intervention proved effective, then we would have discovered a scalable way to motivate learners’ use of effective learning strategies in the real world, through an accessible, online mode that adolescent learners can administer on their own.

As a start to understanding heterogeneity in our intervention’s effects, we tested for potential moderation by individual differences in academic preparedness and students’ perceptions of their classroom norms. These factors could, in theory, be related to the efficacy of intervening on a strategic mindset. Specifically, we used students’ prior academic achievement as a proxy of their prior academic preparedness (which we validated against their self-reported motivation to learn and their learning strategy use at baseline, pre-intervention). We measured students’ perceptions of how much their peers were engaged with the intervention. These are peer norms which reflect the conduciveness of their learning environment for engaging with the intervention, and perhaps also for learning more generally throughout the school year. By exploring such heterogeneity, our work importantly contributes toward calls in the scientific literature to better understand the conditions under which social-psychological intervention effects tend to occur, beyond simply asking whether or not main effects exist^24,43^.

We recruited a total of 1070 Secondary 2–4 adolescents (age range: 13–17 years; 54.1% females; further demographic details are provided in Supplementary Table 3) from 77 classes across 6 public schools that signed on to participate from the start of the academic year. Students were invited by researchers to take the online (intervention/control) exercise in their classrooms. To ensure double-blind random assignment, we used Qualtrics software to randomly assign consented students to either receive our strategic mindset intervention exercise (strategic mindset condition: *N* = 536) or a similarly-structured control exercise (control condition: *N* = 534). The online exercises were approximately 40 min in duration, and of the same length and structure. Some students (*n* = 475) received 1 dose about a month before their final exams (Aug–Sept), and others (*n* = 595) received an additional dose earlier in the year (May).

The strategic mindset intervention was inspired by, and adapted from, the intervention tested in Experiment 3 to be age-appropriate and engaging for this younger audience. We additionally sought feedback through interviews with Singapore Secondary school students to improve its design and relevance.

As an overview, the strategic mindset intervention for Secondary school students involved learning about the common challenges that Secondary school students often face; hearing other students’ testimonials about how they had used the strategic mindset psychology to overcome these challenges, to make progress, and to learn better; reading scientific evidence about effective learning; identifying strategy-eliciting questions that they themselves would find useful asking during their studies; and then committing to even more effective ways of learning for their upcoming final exams (the “Methods” section provides more detailed descriptions of the intervention; Supplementary Note 3 shows example intervention screenshots).

Importantly, we did not train students in the use of specific learning strategies, but instead focused on teaching them the value of asking themselves strategy-eliciting questions when they encountered challenges—the crux of a strategic mindset. Because of technical limitations in some Secondary schools, we chose to convey our messages using slides and interactive, open-ended questions, instead of videos.

To ensure that the control condition was exposed to a topic that was relevant and important to them, students randomly assigned to the control condition learned about the value of co-curricular activities (CCAs), and how to choose and thrive in one that fits them. Co-curricular activities include physical sports (e.g., gymnastics, badminton), clubs and societies (e.g., chess club, computer club), uniform groups (e.g., scouts, girls’ brigade), and visual and performing arts (e.g., modern dance, arts & crafts club). CCAs are a key component of students’ holistic education in Singapore and an important way for students to build socioemotional skills, physical fitness, character and moral values, a sense of belonging, friendships, and more. We kept the structure and duration of the control exercise similar to the strategic mindset intervention, but crucially excluded strategic mindset messaging from the control content.

Immediately after completing the intervention or control exercises, students answered our key manipulation check that assessed their attitude toward a strategic mindset (example item: “If students are struggling with something, they should ask themselves: ‘How else can I approach my learning to be even more effective?’”; 7-point scale). These questions were embedded among other filler survey questions to minimize demand effects.

We measured our theorized mediator (students’ reported use of learning strategies known to be effective for learning) shortly after their final exams at the end of the school year. Using the LASSI Skill subscale previously described in Study 1, students reported what effective learning strategies they had used when studying for their final exams. As our performance outcome measure and with students’ consent, we obtained their final exam performance from their schools at the end of the school year.

To analyze possible heterogeneity of our intervention effects, we assessed students’ prior academic preparedness by obtaining their prior year’s academic performance scores. To validate this, students rated their academic motivation (using the LASSI Will 5-point subscale; example item: “Even if I am having difficulty in a subject, I can motivate myself to complete the work.”) and their use of learning strategies (using the LASSI Skill 5-point subscale described earlier) pre-intervention. Students also reported their perceptions of classroom peer norms regarding engagement with the intervention (survey item: “Consider the students around you. How many students would you say were working carefully and quietly on this activity today?”; 5-point scale, where *1=Fewer than half of students* and *5=All students*).

In our main analyses, we used mixed effects models including random effects for education level and school, as well as class nested within level and class nested within school. This is because school exams were consistently designed and administered across all classes within each education level, and it did not make sense for us to estimate fixed effects of school given the small number of schools.

First, our manipulation of a strategic mindset was effective. Students randomly assigned to the strategic mindset condition reported, on average, more positive attitudes toward adopting a strategic mindset after the intervention, compared to students assigned to the control condition (*b* = 0.12, *t* = 2.12, *p* = 0.035). This result suggests that the intervention message was persuasive. Students’ attitudes toward a strategic mindset (assessed 1 month prior to their final exams) positively predicted their final exam scores (*b* = 0.14, *t* = 5.58, *p* < 0.001), supporting the predictive value of our manipulation check measure.

We tested our pre-registered hypotheses: first, that intervening on a strategic mindset would increase students’ use of effective learning strategies; and second, that students’ randomly assigned condition (strategic mindset vs. control) would affect their final exam performance, mediated through increased use of effective learning strategies.

However, at pre-intervention baseline, the average prior year academic performance of students who were randomly assigned to the strategic mindset condition was significantly lower than that of students in the control condition (*b* = −0.84, *t* = −2.15, *p* = 0.032). Because of this imbalanced randomization, we could not simply conduct our analyses as originally pre-registered. Instead, we included prior year’s academic performance as a covariate to account for this imbalance.

**Fig. 3:**
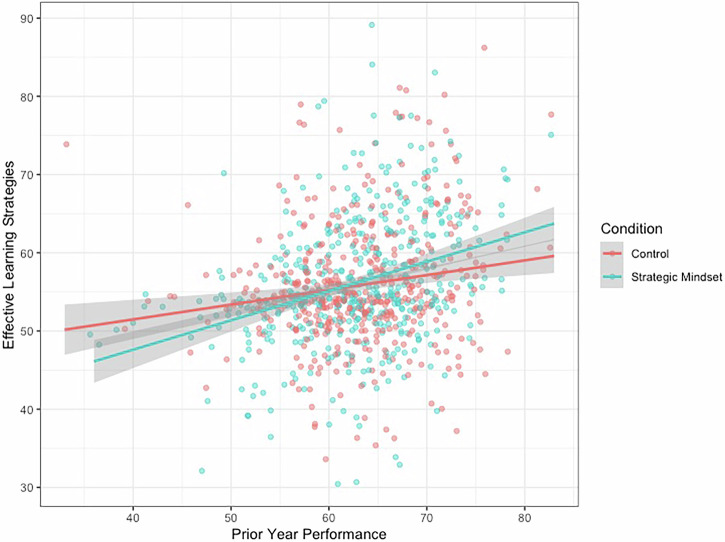
Interaction between condition and prior year’s performance on students’ reported use of effective learning strategies. The figure illustrates the Condition X Prior Year’s Performance interaction on students’ reported use of effective learning strategies when studying for their final exams. The intervention significantly benefitted students who performed at +1 SD above the mean on prior year performance, but the difference was not statistically significant for those who scored at the mean or at −1 SD below the mean on prior performance.

Contrary to our first hypothesis, we did not find an effect of condition on students’ reported use of effective learning strategies, controlling for prior year’s academic performance, *p* = 0.429. However, this was qualified by a significant interaction with their prior performance (interaction *b* = 0.19, *t* = 2.56, *p* = 0.011). Students who performed 1 standard deviation (*SD*) above the mean the year before significantly benefitted from receiving the strategic mindset intervention (simple effect *b* = 1.68, *t* = 2.35, *p* = 0.019); whereas students who performed at the mean or 1 SD below the mean did not perform significantly differently from the control condition (simple effects *ps* > 0.180). Figure 3 visually presents this interaction.

Next, we tested our psychological process model, which is that students’ strategic mindset would impact exam performance through their use of learning strategies. Since we found a significant moderation by prior performance (where higher performing students who received the intervention reported using more effective learning strategies), we conducted a moderated mediation test, using the *Process* function in *R* (model 8^33^) with 1000 bootstrap resamples. To test if the mediation would be significant for higher-performing students, we specified a Condition X Prior Performance interaction predicting students’ use effective learning strategies and their final exam performance. Indeed, we found evidence of moderated mediation, with students’ use of effective learning strategies as a mediator of the relation between condition and final exam performance, and prior performance as a moderator (index of bootstrapped moderated mediation = 0.02 [0.004, 0.04]).

In practical terms, we observed the theorized, significant indirect effect pathway at higher (but not lower) levels of prior performance, suggesting that more (but not less) academically prepared students seem to benefit from this strategic mindset intervention. For instance, if we take an averaged prior exam performance of 70 percentage points (which corresponds to an “A” in the Singapore education system), we find a significant indirect effect of 0.19 [0.01, 0.40]. These students in the intervention condition reported applying more effective learning strategies when studying, and consequently, performing even better on their final exams. As mentioned, the mediation was not significant at lower levels of prior performance.

How might we understand this result? The strategic mindset intervention taught students to ask themselves strategy-eliciting questions, but did not train students in using the specific strategies. Perhaps students who already had an existing repertoire of study strategies could provide useful answers when asking strategic mindset questions. Another possibility is that students who were more motivated to learn and perform well regularly practiced this strategic mindset, which might have been necessary to snowball a brief intervention into effects on their final exams a month later^44^. Indeed, pre-intervention, the students in our sample who had higher prior performance reported already using effective learning strategies to a greater degree (correlation with prior performance: *r* = 0.23, *p* < 0.001) and were more academically motivated (*r* = 0.39, *p* < 0.001). Thus, it is possible that this particular instantiation of a strategic mindset intervention for adolescents speaks to and benefits students who tend to be more academically prepared.

Additionally, we found that perceived peer norms around engaging with the intervention also significantly moderated the psychological process (index of moderated mediation = 0.32 [0.03, 0.62]), even when controlling for prior performance (index of moderated mediation with covariate = 0.12 [0.01, 0.25]). On average, students rated high levels of peer engagement during the intervention (*M* = 3.60, SD = 1.21, mode = 5.0). This suggests that, on average, most to all students in each participating class were working carefully and quietly on their respective intervention or control exercises. As we might expect, the intervention was more effective when students perceived a higher percentage of their peers to be actively engaged with it. There was a significant indirect effect pathway at higher (but not lower) levels of peer engagement. For example, among students who reported perceiving that all other students in their classes were engaged during the study session (average rating of 5.0 on a 5-point scale), we observed a significant indirect effect of 0.57 [0.04, 1.13]. These treated students in conducive peer environments reported applying more effective learning strategies when studying, and consequently, performed better on their final exams. However, the mediation was not statistically significant at lower levels of perceived peer norms.

Overall, we found that this first instantiation of a strategic mindset intervention for adolescents seemed to benefit students who were more academically prepared as well as those in conducive classroom environments with high levels of perceived peer engagement during the study session.

Although we did not pre-register tests for moderation by dosage (i.e., whether students received only 1 session of the strategic mindset intervention or 2 sessions of it), we tested for this in exploratory, secondary analyses. We did not find statistically significant three-way interactions of Condition X Prior Performance X Dosage predicting either students’ learning strategy use (*p* = 0.081) or final exam performance (*p* = 0.578). Therefore, at least in the current study, we cannot conclude that increasing the dosage from one to two sessions has any significant effect on our main outcome variables.

## Discussion

To learn effectively, students can benefit from frequently asking themselves how they might improve their learning. We provide evidence that this strategic mindset predicts how effectively students learn and how well they perform academically. Four correlational and experimental studies on 7475 students demonstrate that, from adolescence to adulthood, those with a strategic mindset tend to apply more effective strategies during learning, and consequently, perform better in school. This mindset matters for learning even in challenging, tumultuous times, and has implications for both school-based exams as well as high-stakes, standardized, national exams. Evidence from two randomized, controlled experiments suggests that cultivating a strategic mindset can be done online in a scalable, self-administered way, and that a relatively brief intervention seems to be efficacious, at least for certain kinds of students under conducive conditions.

Our work extends the ecological validity, experimental evidence, and cultural and developmental generalizability of a strategic mindset beyond prior studies. Evidently, a strategic mindset has important implications for real-world learning and performance. The psychological process through which a strategic mindset operates on learning appears to be generalizable to non-Western samples and from adolescence to adulthood. Crucially, we provide the first scalable, cost-effective strategic mindset intervention that adolescents can self-administer. By doing so, our work advances scientific understanding of how to support strategic, effective learning and achievement in a practically impactful way.

This research makes a theoretical contribution to the literatures on self-regulated learning and metacognition. Across four studies, we deeply examine strategic mindset psychology to address scientific questions (about why some people use more effective strategies than others, and how to intervene in a scalable manner) that complement prior research. In our current experiments and others^23,28^, even when controlling for baseline strategy knowledge or innate self-regulatory abilities, a strategic mindset still accounts for individual differences in the use of effective strategies, and in turn, performance.

Our intervention’s main ingredients focused on motivating students to develop a mental habit of asking themselves strategy-eliciting questions. Even though it did not explicitly teach any specific strategies, the strategic mindset intervention significantly increased students’ reported use of effective learning strategies. Because it may be practically impossible to teach people every specific, effective strategy for every circumstance that they may encounter, cultivating a strategic mindset could instead empower individuals’ autonomous self-regulation. By spontaneously and habitually asking themselves strategic mindset questions, learners can take better control of their own search for, and use of, task-appropriate strategies.

This research paves the way for future strategic mindset interventions that could potentially complement other approaches used in promoting effective strategy use, such as strategy training^20,22,45–51^. Equipping students with a combination of a strategic mindset and explicit training in how and when to use specific, effective strategies may strengthen the impact and durability of our intervention. This combination could be especially effective for lower-performing or less academically prepared students, as well as for adolescents and children, who may lack a basic repertoire of effective learning strategies with which they can answer strategic mindset questions.

In addition to academic preparedness, the external peer environment is also crucial for intervention fidelity and efficacy. Corroborating this, anecdotal evidence from the research team suggests that it may have been difficult for students to pay attention to (let alone engage conscientiously and thoughtfully with) the intervention in some classes, which were more chaotic during the study session. Future field studies could try to increase intervention fidelity during administration or design the intervention in ways that are intrinsically more engaging. At the same time, students’ perceptions of their peers’ engagement may also be reflective of how conducive the classroom is to learning throughout the year. These findings offer insight into the environmental conditions that might be necessary for an online strategic mindset intervention (and perhaps online psychological interventions more broadly) to be effective when self-administered by students in classroom settings.

As previous interventionists have theorized, psychological interventions are like high-quality seeds planted in fertile soil^52^: they grow best in conducive conditions. Such conducive conditions include appropriate affordances in the external context (e.g., adequate and appropriate learning resources, teachers’ support), as well as the intrapersonal, psychological preparedness of the learner (e.g., being equipped with learning skills or feeling motivated to learn). Our field experiment simultaneously demonstrated that both of these factors (a conducive external context and intrapersonal psychological preparedness) can be crucial for psychological intervention efficacy.

These results are promising for a first real-world, field test of a brief, online and scalable intervention that adolescents can self-administer. At the same time, there is certainly a lot of room for future research to build upon. One important direction is to adapt and test the strategic mindset intervention for different student populations in different educational or cultural contexts. We want to develop strategic mindset interventions that better support less-prepared adolescents (who lack a strategic mindset and an accompanying toolbox of effective learning strategies), struggling tertiary-level learners (who may be aware of various learning strategies but are not effectively applying effective ones when needed), and older adults returning to school to advance their skills (who may benefit from reassessing their methods and picking up new strategies for learning). Further testing could better understand how much of the strategic mindset intervention might be universally relevant and what parts of the intervention may need to be tailored to specific groups of people and contexts. Additionally, longitudinal intervention studies or follow-up measurements for our current field intervention could test even longer-term effects of a strategic mindset intervention on students’ academic outcomes and trajectories, as well as on other important outcomes, such as career success or lifelong learning.

For less motivated individuals, it may be useful to combine a growth mindset intervention^42,53^, which cultivates in students the belief that their abilities can be developed, with the strategic mindset intervention. This synergy, which we are exploring in ongoing research [(Teo & Chen, in prep)], could potentially motivate students to believe that they *can* learn and improve through effort, mentorship, and effective strategies (the core idea of a growth mindset), and to also frequently consider *how* they should direct their efforts productively to actualize such growth (the core idea of a strategic mindset).

Here, we measured students’ self-reported use of various learning strategies using a well-validated instrument^4,54^. These self-reports were, in our research and in prior literature^4,8,9^, positively correlated with objective performance outcomes. In prior work, people’s self-reported strategy use also converged with their observed behavioral strategy use (Study 3 from ref. ^23^). The empirical evidence all together give us reason to believe the validity of students’ self-reported learning strategy use in our studies. Nevertheless, future research could adopt more behavioral measures to assess students’ actual strategy use. For example, they might assess learner’s use of online learning resources or their actual changes in problem-solving strategies when working on a challenging science problem in class.

Beyond academic preparedness and peer norms, it could be useful to understand other possible forms of heterogeneity in the intervention’s effects. For example, there could be variation by socioeconomic status or parental education levels, which may reflect differences in how much strategic mindset support and modeling children receive at home. Other classroom-level factors, such as teachers’ explicit strategy instruction or the extent to which they provide students with opportunities to explore various learning strategies, may also moderate the intervention’s impact. Relatedly, our ongoing studies are starting to study how a strategic mindset develops, such as by investigating the role that parents and teachers might play in nurturing a strategic mindset.

After further testing and refinement, strategic mindset interventions could eventually be incorporated into existing curricula to support students’ learning^55^. For example, teachers could encourage and give students opportunities to practice developing a mental habit of frequently asking themselves strategic mindset questions, especially when students are stuck, unproductive, or when they encounter difficulty during learning. Teachers could purposefully cultivate a “strategic mindset culture” in their classrooms—for example, by role modeling the self-questioning process when they are stuck, coaching students in how to develop this mental habit over time, prompting students with strategic mindset questions, and spotlighting students who are practicing the mindset effectively^55–57^. In addition to teachers’ exhortations, teachers and curriculum designers could further scaffold this mental habit among students by embedding strategic mindset questions within textbooks and homework worksheets. Much like metacognitive reflection exercises and feedforward prompts^55^, such external, metacognitive supports may help to motivate and to bolster students’ long-term internalization and use of the strategic mindset.

In conclusion, a strategic mindset supports effective learning and academic performance, even when the going gets tough. This research expands the real-world impact and generalizability of this strategic orientation toward life, offering new ways in which we can support students’ success.

## Methods

Study 1 and Experiments 3 and 4 were approved by the National University of Singapore Institutional Review Board; Study 2 was deemed exempt from IRB review and we received approval to conduct the research. Study 1 and Experiment 4, which involved Primary and Secondary schools, obtained additional approval from the Ministry of Education, Singapore. For Study 1 and Experiment 4 with adolescents, we obtained students’ assent and parental consent to participate; for Study 2 and Experiment 3 with college students, we obtained participants’ consent to participate.

### Study 1

This study data were collected as part of a project funded by a National Research Foundation (Singapore) Fellowship to PI Chen. This research received approval from the institutional IRB and the Singapore Ministry of Education for data collection. We recruited government and government-aided schools (schools that follow the standardized, national syllabus and the standardized school year) in Singapore in 2020. As a reference, there were a total of 179 Primary and 129 Secondary government or government-aided schools in Singapore in 2020. Our study involved 15 Primary schools and 14 Secondary schools (29 participating schools in total).

We recruited by first directly reaching out to school leaders (e.g., Principal, Vice Principal). With leadership approval, we subsequently worked with a school representative within each school to disseminate parental consent forms to participating classes. Students whose parents gave informed consent were allowed to participate in our surveys if they too assented to participate in each survey. We offered students bookstore vouchers for their participation. Students were asked for their assent at the start of each of our online surveys. They took the surveys online on their own via Qualtrics in class or at home (which we accommodated by request from some schools because of the disruptions during the pandemic, especially during the mid-year period).

In Singapore, the academic year for Primary and Secondary schools corresponds with the calendar year, running from January to December. In the larger project within which our data collection was embedded, there were 3 survey waves during the 2020 academic year: Time 1 occurred at the beginning of the school year (Feb–March; Term 1); Time 2 in the middle of the school year (July–August; Term 3) before their final exams; Time 3 at the end of the school year mostly after students’ final exams or national exams (September–early November; Term 4). For this paper, we focus on Time 1 and Time 2 that are directly relevant to our hypothesis-testing.

The Covid-19 pandemic started to impact teaching and learning in 2020 when this study was conducted. Notably, students were still going to classes in schools as per normal at the start of the school year (the time when we administered our Time 1 survey with the strategic mindset scale suitable for adolescents). However, after that, at the height of the pandemic, schools in Singapore underwent a nationwide, mostly centrally coordinated transition to fully online learning. Schools then transitioned back to in-person classes by Term 3. At this mid-point of the school year (Time 2), we collected our measure of learning strategy use by asking students to report their use of various effective learning strategies using the LASSI. We describe each of our scale measures and performance outcome variables in further detail below.

To create our strategic mindset scale for adolescents, we adapted the items from the original strategic mindset scale for adults^23^ to be appropriately worded for adolescents. The 4-item scale reliability was high: *α* = 0.88. The items included the following:

(1 = Never, 5 = Most of the time)

1. When something is very hard for you, how often do you ask yourself: “What can I do to make myself better at this?”
2. When you are struggling with something, how often do you ask yourself: “What can I do to help myself?”
3. When you are stuck on something, how often do you ask yourself: “What are other ways I can think of trying?”
4. Whenever you feel like you are not making progress, how often do you ask yourself: “Is there a different way I can do this?”

To measure students’ reported use of learning strategies, we used the LASSI Skill subscale from the LASSI (3^rd^ Edition College version^4^), which encompasses students’ learning, study, and test-preparation strategies. This version was recommended by the publishers, H&H Publishing, for our purposes. The scale measures “students’ learning strategies, skills, and thought processes related to identifying, acquiring and constructing meaning for important new information, ideas and procedures, and how they prepare for and demonstrate their new knowledge on tests or other evaluative procedures” (ref. ^4^, p. 7-8). We included all 3 Skill subscale components, including information processing, selecting main ideas, and test strategies. The LASSI Skill subscale showed high reliability: *α* = 0.85. We did not use the Will or Self-Regulation subscales, because this paper focuses on linking a strategic mindset to students’ use of cognitive strategies.

Due to copyright, we are unable to share the full measure. However, we have described some example items earlier. Interested parties may contact H&H Publishing, which holds the copyright permissions, to get access to the full measure. Of note, we minorly adapted some of the item wording to be appropriate for Singapore students (e.g., changing the term “assignment” to “homework” or the term “review” with “go through,” which is more understandable for Singapore Primary and Secondary school students). We kept the main parts and goals of the items the same for construct precision.

With parental consent and students’ assent, we obtained students’ year-end examination scores as our performance outcome of interest. These year-end school examinations are set by each school independently, although schools in Singapore tend to closely follow the curriculum set by the Singapore Ministry of Education. Note that the grading scale used in Singapore tends to follow the British system (also used in other parts of the world), where 70% corresponds to an A. For each examinable subject that students took (e.g., languages, mathematics), students’ scores were converted to a score out of 100 percentage points, and then averaged across all the examinable subjects that students took. This allows us to have a comparable measure across grade levels that is independent of the number of subjects that a student is taking.

In addition to school exams, we also obtained students’ national exam scores, where applicable. There were two important National Examinations: the Primary School Leaving Examination (PSLE) taken in the final year of Primary School (Primary 6), and the GCE O-Levels or N-Levels, taken in Secondary 4 (approximate ages: 15–16). These examinations are standardized across the whole country—that is, every student takes the same exam paper, so results are comparable across schools. Student performance on these exams matters for their academic trajectories, such as determining the schools and subjects that they are eligible for at the next level.

In 2020 (the year we collected our data) and prior, the PSLE used a “*T*-score”—a standardized score measuring how well a student does relative to their peers across the whole country. Students are tested in 4 subjects: English, Mother Tongue, Mathematics, and Science. The *T*-score is based on the sum of the standardized scores that students obtain in the 4 subjects, along with an affine transformation to keep the values human-readable. Please refer to the Supplementary Note 1 for details about the mathematical calculation of the *T*-score. For the purposes of comparing with the rest of our performance variables (e.g., Table 1), which are out of 100 percentage points, we divided the *T*-score by 3. We did not do any additional transformation, because this variable is already normally distributed. This rescaled variable has a mean of 69 (SD = 11.6), a median of 70, and a range of 24–91. This is comparable to the year-end school final exam performances: mean 63 (SD = 11.5), median 63, range of 0–96.

In Secondary School in Singapore in 2020, students in our target sample were either in the “Express” stream (who take the O-Levels in Secondary 4) or the “Normal (Academic)” stream (who take the GCE N-Levels in Secondary 4, with the option to take the GCE O-Levels in Secondary 5 instead). There are both “Express” and “Normal (Academic)” students in our sample, so we present analyses with both National O-Level (Secondary 4 Express) and N-Level (Secondary 4 Normal (Academic)) results. We did not have any Secondary 5 Normal (Academic) students in our sample. Some student participants (less than 10%) indicated that they were in an Integrated Programme, which allows some Secondary school students to skip the GCE O-Levels exam in Secondary 4. In our analyses with national exam performance, we excluded analyses on this small subset of students who did not take the national exams.

Each subject in the GCE O-Levels and N-Levels is graded by bands, and is scored from best to worst: A1, A2, B3, B4, C5, C6, D7, E8, and F9. The numerical score for the 6 best-performing relevant subjects in the O-Level exams, and the 5 best-performing relevant subjects in the N-Level exams, are summed, to give the student’s overall score. The “relevant” subjects for the O-Level exams (“L1R5”) include: English or a language at the first-language-learner level, at least one mathematics subject, at least one science subject, and at least one humanities subject. The “relevant” subjects for the N-Level exams (“ELMAB3”) include: English, Mathematics, and the best three subjects.

Thus, in the O-Level exams, with 6 subjects, the best possible score one can get is 6 (which entails scoring an A1 for all their relevant subjects), and the worst possible score is 54 (which entails scoring an F9 for all their relevant subjects). For the N-Level exams, the best and worst possible scores are 5 and 45, respectively, as the N-Level exams score only counts 5 subjects. The mean O-Level exams score in our sample was 18.9 (SD = 7.8), while the mean N-Level exams score in our sample was 14.6 (SD = 3.83). For interpretability and consistency across all the different exams and education levels, we transformed these national exam scores into their percentage points equivalent for our main analyses. Please refer the Supplementary Note 1 for details about how we transformed these raw scores into their percentage point equivalent using an affine transformation. We present the results of these transformed percentage point scores in Table 1 and the results using the raw scores in Supplementary Table 2.

Of note, when testing our hypothesized psychological process, we observed consistent results across school exam scores (Table 1), raw national exam scores (Supplementary Table 2), and transformed national exam scores (Table 1).

### Study 2

This study was pre-registered at as.predicted.org (#119308). As mentioned, this cross-sectional survey was conducted in 2021 when students were enrolled in online, in-person, and/or hybrid classes that were structured to accommodate the pandemic. These conditions offered a fortuitous opportunity to test the effectiveness of students’ learning amidst such challenging conditions. We describe each of the scale measures in further detail below.

We used the strategic mindset scale developed for adults from ref. ^23^. The scale reliability was high: *α* = 0.93. The items included the following:

(1 = Never, 5 = Most of the time)

1. In moments when you feel challenged, how often do you ask yourself: “What are things I can do to make myself better at this?”
2. When you are struggling with something, how often do you ask yourself: “What can I do to help myself?”
3. Whenever something feels difficult, how often do you ask yourself: “What can I do to get better at this?”
4. When you are stuck on something, how often do you ask yourself: “What are things I can do to help myself?”
5. Whenever you feel like you are not making progress, how often do you ask yourself: “Is there a better way of doing this?”
6. Whenever you feel frustrated with something, how often do you ask yourself: “How can I do this better?”

We used the LASSI Skill subscale (3^rd^ Edition College version^4^), which encompasses students’ learning, study, and test-preparation strategies. We used the original college version, since the wording was already appropriate for university-level students. As mentioned earlier, due to copyright, we are unable to share the full measure, but we have provided an example item earlier. Interested parties may contact H&H Publishing, which holds the copyright permissions, to get access to the full measure. The LASSI Skill subscale showed high reliability in our sample: *α* = 0.87.

### Experiment 3

We describe each of the key variables measured here. First, as a manipulation check, we wanted to assess whether, compared to the control condition, our strategic mindset intervention would be effective at increasing students’ positive attitudes toward adopting a strategic mindset. We could not use the original strategic mindset scale as a manipulation check measure immediately post-intervention, because it is a behavioral frequency scale that asks how much students tend to practice the strategic mindset in general, and we would not expect to detect changes on it immediately post-intervention. Hence, we measured students’ attitudes toward adopting a strategic mindset as our manipulation check. The items included the following:

(1 = Strongly disagree; 7 = Strongly agree)

1. Whenever I feel challenged, I should always ask myself: “What can I do to help myself?”
2. Whenever I struggle with something, I should always ask myself: “How else can I do this?”
3. Whenever something feels difficult, I should always ask myself: “What are things I can do to make myself better at this?”
4. Whenever I am stuck, I should always ask myself: “Is there an even better way of doing this?”
5. Whenever I feel frustrated, I should always ask myself: “What else can I do differently?”
6. Whenever I feel like I am not making progress, I should always ask myself: “What else can I do differently?”

We interspersed these items with other unrelated filler items to reduce demand effects. The 6 items were internally consistent (*α* = 0.85), so we averaged them into a single composite.

In addition to assessing students’ attitudes, we measured their self-reported intentions to use a strategic mindset during learning in the future. Since research shows that intentions are an antecedent to behavior^40^, we wanted to know if self-administering the intervention would have an effect on students’ intentions to apply a strategic mindset in the future. We instructed students to report how likely they were going to do the following over the upcoming few weeks:

(1 = Not at all; 7 = Extremely likely)

1. Whenever I am stuck, I will ask myself whether there are other ways to approach the problem.
2. Whenever I am struggling with something, I will ask myself whether there are other ways to try it.
3. Whenever something feels difficult, I will ask myself what I can do to help myself get better.
4. Whenever I feel challenged, I will ask myself whether there are things that I can do to help myself.
5. Whenever I feel like I am not making progress, I will ask myself if there is a better way of doing things.
6. Whenever I feel frustrated with something, I will ask myself to think about what else I can do.

We interspersed these items with other unrelated filler items to reduce demand effects. The 6 items were internally consistent (*α* = 0.93), so we averaged them into a single composite.

To measure students’ future intentions to use effective learning strategies, we used a prospectively-worded version of the LASSI Skill subscale described in Study 2, by changing the item wording and instructions to reflect students’ future intentions to use each of the learning strategies. Our instructions were: “In the next 2 weeks, how frequently will you commit to doing the following when studying?” To keep the measure concise, we included 6 items that specifically measured students’ cognitive learning and study strategies. Some examples of our items included:

(0% = Not at all in the next 2 weeks; 50% = About half the time when studying in the next 2 weeks; 100% = All the time when studying in the next 2 weeks)

1. Even when there are many details to read in my textbooks and readings, I will identify the important information I need to remember.
2. Whenever I listen to class lectures, I will ask myself what information is important to know.
3. Whenever I’m learning, I will try to find relationships between what I am learning and what I already know.
4. Whenever I’m learning, I will translate what I am studying into my own words.

The items were internally consistent (*α* = 0.85), so we averaged them into a single composite measure to reflect students’ future intentions to use effective learning strategies.

To test that our strategic mindset intervention specifically and uniquely changes a strategic mindset, and not a growth mindset, we included a measure of the growth mindset of intelligence^58^. We used a 7-point response scale to also include a “neutral” midpoint, instead of the original 6-point response scale. The items included the following:

(1 = strongly disagree; 7 = strongly agree)

1. You can learn new things, but you can’t really change your basic intelligence.
2. Your intelligence is something about you that you can’t change very much.
3. You have a certain amount of intelligence and you really can’t do much to change it.

The items were internally consistent (*α* = 0.87), so we averaged them into a single composite.

### Experiment 4

This experiment was part of a larger data collection, which included other experiments and hypotheses testing. For the purposes of this paper, we focus on the intervention conditions that have strategic mindset messaging for comparison against a control condition. We included in our analyses 6 out of 7 schools who agreed to participate from the beginning of the academic year, excluding one school that only officially agreed to take part midway through the school year after testing in other schools was already underway.

In the strategic mindset intervention, we shared with students the common challenges that many Secondary school students often face, such as encountering difficulty in some subjects, struggling to understand everything in class, getting stuck on their homework problems, or feeling disappointed with their exam grades. Crucially, we emphasized that many students say that they want to learn how they can overcome these challenges and to improve. This content was informed by previous surveys and interviews that we had conducted with other Secondary school students. Next, students learned that those who learn effectively and succeed at overcoming challenges tend to think about what they are learning and to ask themselves strategy-eliciting questions, such as “How can I learn even better?” Moreover, these students do not just study *hard* (by putting in a lot of time), but they also study *smart*—by using effective study methods.

Students then read stories from other Secondary school students like them—about how these students had similarly experienced challenges, asked themselves strategy-eliciting questions in those moments, and then used those opportunities to find even better ways of learning. These student stories were tailored from real stories that were shared with us during student interviews and through earlier surveys. Students also read about scientific facts that supported our arguments.

Throughout the intervention, students were asked questions to help them internalize, personalize, and apply what they had learned. For example, they identified which strategy-eliciting questions they would find helpful to ask themselves when studying (e.g., “What can I do to help myself learn this well?”, “Are there other, even better ways to learn this?”), and then wrote down their answers to these questions by thinking through, and committing to, even better ways of learning for their final exams. Supplementary Note 3 provides example screenshots from the strategic mindset intervention. Next, we describe the key scale measures and performance variables used.

As a manipulation check, we wanted to assess whether, compared to the control condition, our strategic mindset intervention would be effective at increasing students’ positive attitudes toward adopting a strategic mindset. Hence, we measured students’ attitudes toward adopting a strategic mindset. As mentioned in Experiment 3, we did not use the original strategic mindset scale as a manipulation check measure immediately post-intervention, because it is a behavioral frequency scale on which we would not expect to detect changes immediately post-intervention. Therefore, right after our intervention, we used this attitudinal measure toward adopting a strategic mindset instead, as an indication of the extent to which our intervention message about using a strategic mindset was persuasive:

(1 = Strongly disagree; 7 = Strongly agree)

1. If students are learning something that feels very hard, they should ask themselves: “What can I do to help myself learn this well?”
2. If students are struggling with learning something, they should ask themselves: “How else can I approach my learning to be even more effective?”
3. When students feel like they are not making progress in their studies, they should ask themselves: “Are there other, even better ways to learn this?”
4. When students feel stuck when doing their homework, they should ask themselves: “Is there a different way to solve this problem?

The items were internally consistent: *α* = 0.90. We averaged them into a single composite. To minimize demand effects, we also included filler items among these scale items.

To measure students’ reported use of learning strategies, we administered similar items from the LASSI Skill subscale as reported in Study 1. We measured this once pre-intervention (to validate students’ academic preparedness pre-intervention), as well as post-intervention after students’ final exams (as our outcome measure of learning strategy use and hypothesized mediator of the indirect effect of condition on performance). As mentioned, interested parties may contact H&H Publishing, which holds the copyright permissions, to get access to the full measure. The items were internally consistent pre-intervention (*α* = 0.80) and post-intervention (*α* = 0.76).

To measure students’ academic motivation pre-intervention, we minorly adapted the “Will” motivation subscale from the LASSI (3^rd^ Edition College version). The purpose of this was to validate students’ academic preparedness pre-intervention. The scale measures “students’ diligence, self-discipline, and willingness to exert the effort necessary to successfully complete academic requirements” (p. 9, ^4^). Due to copyright, we are unable to share the full measure. Interested parties may contact H&H Publishing, which holds the copyright permissions, to get access to the full measure. The 6-item motivation subscale showed good internal consistency: *α* = 0.77.

To test for possible heterogeneity of the intervention effects by prior academic preparedness, we obtained students’ prior year’s achievement scores from schools as a proxy measure of their overall academic preparedness. To validate this, we additionally measured students’ baseline use of learning strategies (using the aforementioned LASSI Skill subscale) and reported academic motivation (using the aforementioned LASSI Will subscale) earlier in the school year before the intervention was administered. We expected students’ use of learning strategies and academic motivation at the beginning of the school year to be correlated with their prior year’s performance, as indicative of their overall academic preparedness and inclination toward academics.

To test for possible heterogeneity of the intervention effects by perceived peer norms, we measured students’ perceptions of their peer norms around engaging with the intervention using the following item: “Consider the students around you. How many students would you say were working carefully and quietly on this activity today?” (5-point scale, where *1=Fewer than half of students* and *5=All students*).

## Supplementary information

Supplementary Information

## Data Availability

The pre-registrations (for Study 2 and Experiment 4), as well as the Experiment 3 dataset, are available in the OSF repository: https://osf.io/ugbtw/?view_only=a2ea6cca22864889937938c074d364a1. The Study 1, Study 2, and Experiment 4 datasets are not publicly available due to the confidential nature of the students’ performance data, and our agreements with the Ministry of Education, Singapore, and the National University of Singapore. Permission to access the data should be directed to and will require approval from the Ministry of Education, Singapore (for Study 1 and Experiment 4) as well as the National University of Singapore (for Study 2).

## Code availability

The R code and analyses output for each study are available in the OSF repository: https://osf.io/ugbtw/?view_only=a2ea6cca22864889937938c074d364a1.
