# Supplementary material for: A strategic mindset predicts and promotes effective learning and academic performance
Source: NPJ Sci Learn. 2025 Oct 31;10:74. doi: 10.1038/s41539-025-00367-6 (PMC12578917; doi:10.1038/s41539-025-00367-6)

# Supplementary Information for "A Strategic Mindset Predicts and Promotes Effective Learning and Academic Performance"

## Table of Contents


| Table/Section | Contents | Page |
|---|---|---|
| Supplementary Table 1 | Demographics of the Student Participants in Study 1 | 2 |
| Supplementary Table 2 | Indirect Effect of a Strategic Mindset on Final and Raw National Exam Performance (With Use of Effective Learning Strategies as Mediator) Across Schools, Broken Down by Grade Level | 3 |
| Supplementary Table 3 | Demographics of the Student Participants in Experiment 4 | 4 |
| Supplementary Note 1 | Additional Analytical Details for Study 1 and Experiment 4 | 5 |
| Supplementary Note 2 | Example Screenshots of Online Strategic Mindset Intervention Tested on University Students (Experiment 3) | 9 |
| Supplementary Note 3 | Example Screenshots of Online Strategic Mindset Intervention Tested on Secondary School Students (Experiment 4) | 14 |

**Supplementary Table 1: Demographics of the student participants in Study 1.**

| Category | Sub-Category | Frequency (%) | Mean Age (SD) |
|---|---|---|---|
| Grade Level | Primary 6 | 1,381 (26.6) | 11.59 (0.39) |
| | Secondary 1 | 953 (18.4) | 12.55 (0.41) |
| | Secondary 2 | 899 (17.3) | 13.57 (0.38) |
| | Secondary 3 | 1,092 (21.1) | 14.60 (0.43) |
| | Secondary 4 | 860 (16.6) | 15.59 (0.49) |
| Gender | Male | 2,009 (38.8) | |
| | Female | 3,169 (61.1) | |
| | Not reported | 7 (0.1%) | |
| Race/Ethnicity | Chinese | 3,494 (67.4) | |
| | Indian | 588 (11.3) | |
| | Malay | 613 (11.8) | |
| | Others | 425 (8.2) | |
| | Not reported | 65 (1.3) | |

This table shows the demographic breakdown (by grade level, gender, and race/ethnicity) of the student participants in Study 1.

**Supplementary Table 2: Indirect effect of a strategic mindset on raw national exam performance (with use of effective learning strategies as mediator) aggregated across schools, broken down by grade level in Study 1.**

| Performance Outcome by Grade Level | Sample size, *N* | Indirect effect, *ab* | 95% CI |
|---|---|---|---|
| Primary 6 PSLE National Exam, t-score | 873 | 5.91 | [4.60, 7.35] |
| Secondary 4 GCE National Exam (‘O’ levels), raw score | 371 | -0.51 | [-0.93, -0.15] |
| Secondary 4 GCE National Exam (‘N’ levels), raw score | 99 | -0.55 | [-1.04, -0.17] |

This table shows the indirect effect *ab* (with 95% confidence interval) estimates of a strategic mindset on raw examination performance scores at each grade level. The mediator in the model is students’ reported use of effective learning strategies. The main difference from Table 1 in the main text is that here we report the raw results for the National Examinations, specifically t-score for the PSLE; L1R5 for the ‘O’ levels; ELMAB3 score for the ‘N’ levels. Note that lower scores for both the GCE examinations denote higher performance for the ‘O’ and ‘N’ level exams (see Supplementary Note 1 for a detailed description). Primary 6 and Secondary 4 correspond to U.S. Grades 6 and 10. All the indirect effects for each level are statistically significant, which can be inferred from the 95% confidence intervals. Sample size *N* gives the sample size for that analysis after pairwise deletions for missing data (e.g., if we were unable to get their year-end or national exam scores).

**Supplementary Table 3: Demographics of the student participants in Experiment 4.**

| Category | Sub-Category | Frequency (%) | Mean Age (SD) |
|---|---|---|---|
| Grade Level | Secondary 2 | 524 (49.0) | 13.8 (0.43) |
| | Secondary 3 | 475 (44.4) | 14.9 (0.63) |
| | Secondary 4 | 71 (6.6) | 15.8 (0.38) |
| Gender | Male | 491 (45.9) | |
| | Female | 579 (54.1) | |
| Race/Ethnicity | Chinese | 730 (68.2) | |
| | Indian | 97 (9.1) | |
| | Malay | 177 (16.5) | |
| | Others | 59 (5.5) | |
| | Not reported | 7 (0.7) | |

This table shows the demographic breakdown (by grade level, gender, and race/ethnicity) of the student participants in Experiment 4.

## Supplementary Note 1

## Additional Analytical Details for Study 1 and Experiment 4

### Study 1 Additional Analyses Details

#### *Performance Outcome Variable: National Examination Scores*

The PSLE, in 2020 (the year we collected our data) and prior, used a "T-score", a standardized score measuring how well a student does relative to their peers across the whole country. Students are examined in 4 subjects: **E**ng**l**ish, **M**other **T**ongue, **Ma**thematics, and **Sc**ience. The T-score is based on the sum of the standardized scores that students obtained in the 4 subjects, along with an affine transformation to keep the values human-readable.

$$T = 200 + 10\,\frac{x_{EL} - \mu_{EL}}{\sigma_{EL}} + 10\,\frac{x_{MT} - \mu_{MT}}{\sigma_{MT}} + 10\,\frac{x_{MA} - \mu_{MA}}{\sigma_{MA}} + 10\,\frac{x_{SC} - \mu_{SC}}{\sigma_{SC}}$$

Thus, T-scores are centered around 200 and increase (decrease) by 10 for each standard deviation a student scores above (below) the mean for each of the 4 subjects. For the purposes of comparing with the rest of our performance variables (e.g., manuscript Table 1), which are out of 100 percentage points, we divided the T-score by 3. We did not do any additional transformation, as this variable is already normally distributed. This rescaled variable has a mean of 69 (SD = 11.6), a median of 70, and a range of 24–91. This is comparable to the year-end school final exam performances: mean 63 (SD = 11.5), median 63, range of 0–96.

In Secondary School in Singapore in 2020, students in our target sample were either in the "Express" stream (who take the 'O' Levels in Secondary 4) or the "Normal (Academic)" stream (who take a less challenging version called the GCE 'N' Levels in Secondary 4, with the option to take the GCE 'O' Levels in Secondary 5 instead). There are both "Express" and "Normal (Academic)" students in our sample, so we present analyses with both National 'O' level (Secondary 4 Express) and 'N' level (Secondary 4 Normal (Academic)) results. We did not have any Secondary 5 Normal (Academic) students in our sample. Some student participants (less than 10%) indicated that they were in an Integrated Programme, which allows some Secondary school students to skip the GCE 'O' Levels exam in Secondary 4. In our analyses with national exam performance, we excluded analyses on this small subset of students who did not take the national exams.

Each subject in the GCE 'O' Levels and 'N' Levels is graded by bands, and is scored from best to worst: A1, A2, B3, B4, C5, C6, D7, E8, and F9. The numerical score for the 6 best-performing relevant subjects in the 'O' Levels, and the 5 best-performing relevant subjects in the 'N' Levels, are summed, to give the student's overall score. Thus, in the 'O' Levels, with 6 subjects, the best possible score one can get is 6 (which entails scoring an A1 for all their relevant subjects), and the worst possible score is 54 (which entails scoring an F9 for all their subjects). For the 'N' Levels, the best and worst possible scores are 5 and 45 respectively, as the 'N' level score only counts 5 subjects.

The “relevant” subjects for the ‘O’ levels (“L1R5”) include: English or a language at the first-language-learner level, at least one mathematics subject, at least one science subject, and at least one humanities subject. The “relevant” subjects for the ‘N’ levels (“ELMAB3”) include: English, Mathematics, and the best three subjects. The mean ‘O’ level score in our sample was 18.9 (SD = 7.8), while the mean ‘N’ level score in our sample was 14.6 (SD = 3.83).

To assist in interpretation, as the ‘O’ and ‘N’ level scores are not “standardized”, and a lower score is better, we transformed them into a “percentage point equivalent” by applying the following affine transformation:

*Transformed_Score* = (*Worst_possible_score* – **score**) / (*Worst_possible_score* – *Best_possible_score*).

Thus, a student who scores the best possible score would have a rescaled score of 100, while a student who scores the worst possible score would get 0. After this affine transformation, the mean ‘O’ level percentage point equivalent score is 73.2 (SD = 16.2, median = 75.0, range = 20.8 – 100), while the mean ‘N’ level percentage point equivalent score is 76.0 (SD = 9.6, median = 63.2, range = 57.5 – 97.5).

For comparison, students’ mean year-end exam performances, which could differ among schools because each school had the freedom to set their own school exams, was 62.7 (SD = 8.2, median = 63.2, range = 37.0 – 85.6) for the students in the Express stream (who also sat for the national ‘O’ Levels), and 57.3 (SD = 9.2, median = 57.3, range = 37.2 – 82.8) for students in the Normal (Academic) stream (who also sat for the national ‘N’ Levels). One important difference between the scoring systems is that the school exams are graded out of 100 percentage points (or raw points converted to percentages), while the ‘O’ and ‘N’ level results are “banded”—i.e., a score of 75 and above translates to an A1, the best possible score. Although it is uncommon for students to get perfect or near-perfect scores on school exams, many students can and do score above a 75, which translates into the maximum possible ‘O’/‘N’ level transformed score for that subject (100%). Nevertheless, the main point of our analyses in this paper for both the national and the school exams is to test how the strategic mindset predicts exam performance through students’ use of effective learning strategies. When testing this psychological process, we observed consistent results across their school exam scores (main text Table 1), raw national exam scores (Supplementary Table 2), and transformed national exam scores (main text Table 1), despite the difference in means.

***Analytical Approach for Mixed Effects Modeling***

Because of the nested nature of our data, we used mixed-effects linear models to analyze our data, using the *lme4* package in R. Students are in only one class, classes are nested within grades and within school, and grade-levels and schools are crossed, as shown below.

*Nested Structure of Data for Study 1.*

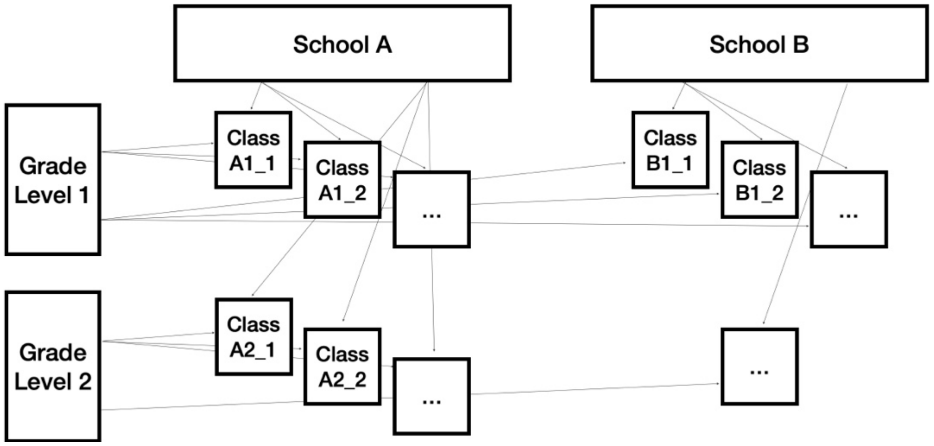


For this reason, we used mixed-effects models with random effects by grade-level, school, and class, to analyze the effect of strategic mindset on learning strategies (LASSI) and on final exam performance. For example, using the lme4 syntax, our model predicting exam score using the strategic mindset would be specified as:

lmer(exam ~ sm + (1|level) + (1|school) + (1|class:school) + (1|class:level))

**Experiment 4 Additional Analyses Details**

***Mixed Effects Modeling***

As explained in the main text, we used mixed effects models including random effects for education level and school, as well as class nested within level and class nested within school:

lmer(exam ~ strategic_mindset + (1|level) + (1|school) + (1|class:level) + (1|class:school))

Here is an example of our mixed effects models that tested for the interaction with prior performance:

lmer(learning_strategy_use ~ prior_performance*condition + (1|level) + (1|school) + (1|class:level) + (1|class:school))

We note that adding academic stream (which in Singapore tends to divide students into a higher-performing “Express” stream versus a lower-performing “Normal” stream by performance) as a fixed effect to the models also produced the same pattern of results.

***Testing Mediation***

To test mediation, we used the *Process* macro in *R* (Hayes, 2022) with 1000 bootstrap resamples. All mediation analyses were conducted at the level of the individual student. We

applied model 8 (in *Process*) in our moderated mediation tests, which specifies moderation of both the x-to-y and x-to-m pathways.

## Supplementary Note 2

## Example Screenshots of Online Strategic Mindset Intervention

## Tested on University Students (Experiment 3)

Through an online video, we shared with students persuasive anecdotes from the lives of highly successful people (e.g., famous athletes, businesspeople, scientists) who exemplified a strategic mindset. We asked them interactive questions about the intervention content afterward, to check their comprehension as well as to give them feedback about the key takeaways of the video.

**Let's revisit what these successful people said.**

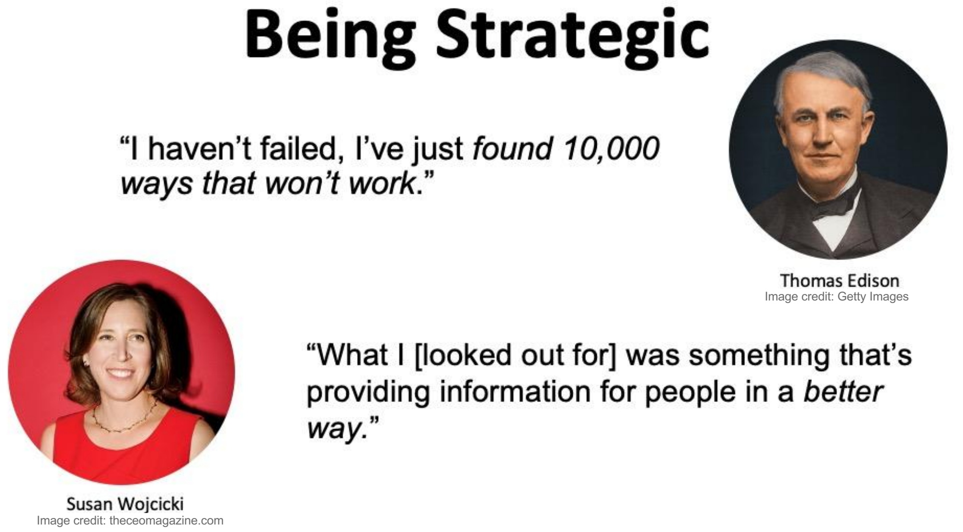


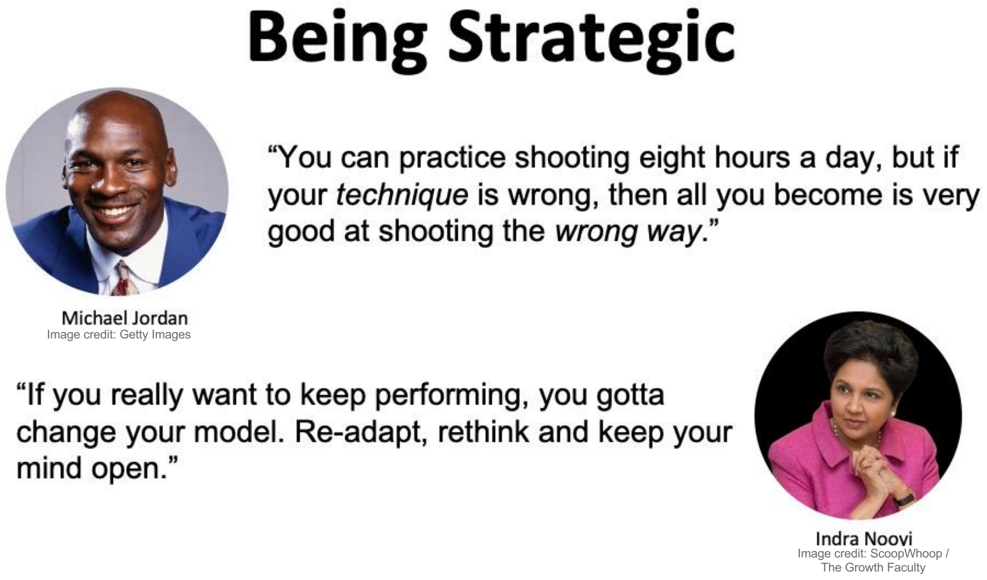

**Successful people are strategic. What this means is that when they encounter challenges, difficulties or new situations, successful people ask themselves:**

- **What can I do to help myself?**
- **How *else* can I do this?**
- **How can I do this *even better*?**

**This is called a strategic mindset.**

We shared insights from scientific research about the importance of this mindset.

Researchers from NUS and Stanford University have found that…

The more people have this strategic mindset,

- the **higher their academic achievements** in university,
- the **more progress they report making towards important health, fitness, and professional goals,**
- the **better they perform on new challenges.**

We recapped what a strategic mindset entailed. We supported these with examples, such as how students can apply a strategic mindset when they are stuck or when they encounter difficulty.

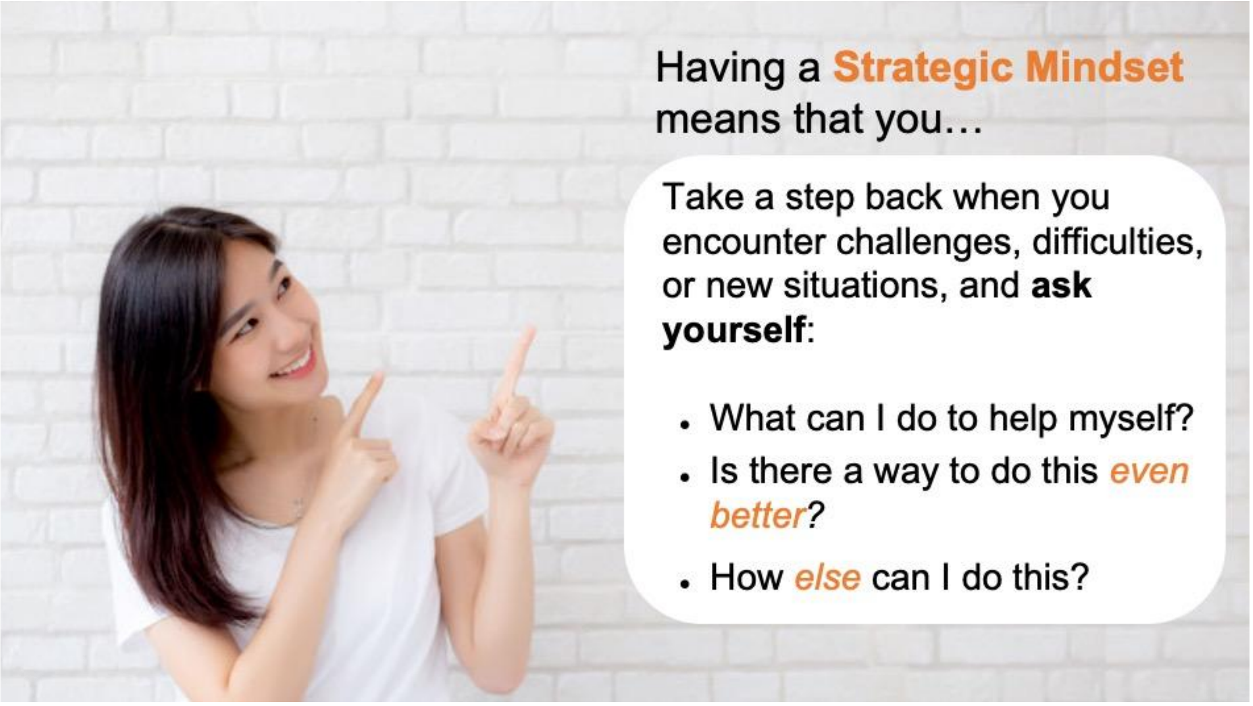


Image credit: ATLANTIS

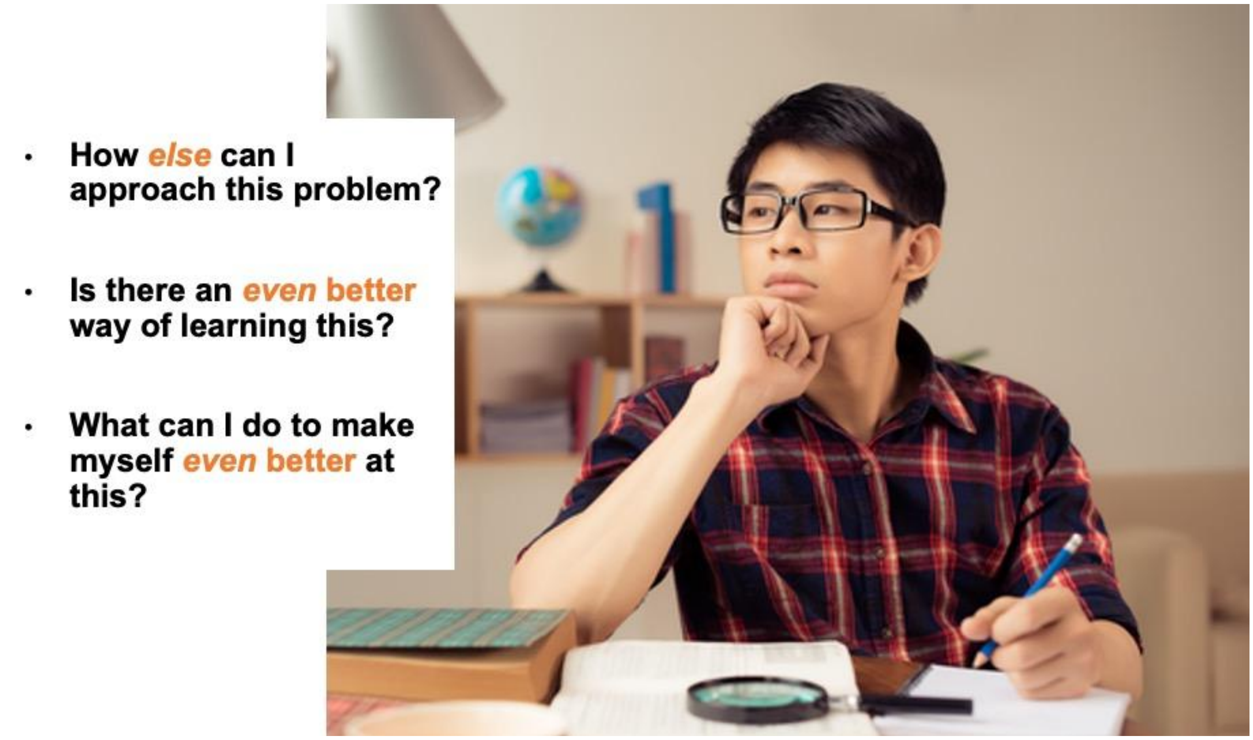


Image credit: C2 Education

With the help of university students, who offered examples and their experiences, we provided examples of what students might say to practice a strategic mindset.

"Have you ever spent some time reading something, only to realise that you don't know what you have just read? Yeah, I hate that. I was studying with my friends the other day but nothing was sinking in. As I was feeling frustrated with myself for being unproductive, the advice I learnt sprung to my mind. I remembered that we need to be strategic about how we studied, and I hadn't been strategic at all. I took a minute to ask myself, **"What can I do differently? Are there other ways that I can learn better?"** Asking these questions made me realise that studying with my friends was unproductive because we were mostly chatting about other things. The next week onwards, I tried studying on my own. I found a good study area that was free from distractions and started studying three times a week. Also, instead of rehearsing information repeatedly when I read from my textbook, I drew figures and tables to better organise my information. Doing this really helped me recall the facts better for my exams. Asking myself, "What can I do differently?" and reflecting on what I wasn't doing strategically helped me study better- and even do better on my tests!

## Supplementary Note 3

## Example Screenshots of the Online Strategic Mindset Intervention

## Tested on Secondary School Students (Experiment 4)

We invited students to help give input into developing a program for future secondary school students. Students learned about common challenges that many Secondary school students like them often face. They also learned that many of their peers expressed a desire to overcome these challenges and improve.

**We are developing a new programme for next year's secondary school students. We would like to invite you to help improve it. This programme will share information about how we can help future secondary school students with their learning challenges and struggles. For example:**

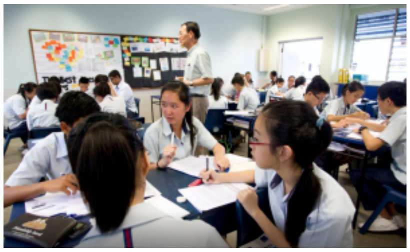

Image credit: The Conversation / Ray Chua / AP / Press Association Images

- **Some students have told us that they find certain subjects difficult in secondary school.**
- **They can't understand everything in class or they get stuck on homework problems.**
- **Others tell us that they feel disappointed when they get back their exam grades.**
- **Many students say that they want to learn how they can improve.**

They learned that students who learn effectively and succeed at overcoming challenges tend to think about their learning approaches and to ask themselves strategy-eliciting questions, such as “How can I learn even better?” Moreover, these students do not just study *hard* (by putting in a lot of time), but they also study *smart*—by using effective study methods.

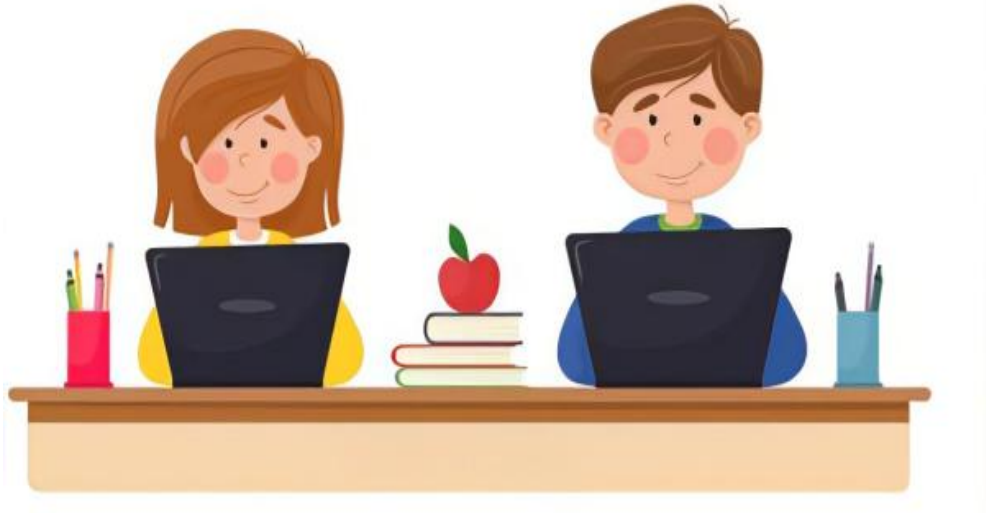

**It turns out that there are some common ingredients that can explain students' learning and progress!**

**1) First, they think about what they are learning (especially if it's hard) and they ask themselves "How can I learn *even better*?"**

**2) They don't just study hard (by putting in a lot of time), but they also study *"smart"* by using good study methods.**

Students read stories shared by other Secondary school students like them. The stories described how those students had experienced challenges and, in those moments, asked themselves strategy-eliciting questions. They then used those moments as opportunities to find even better ways of learning.

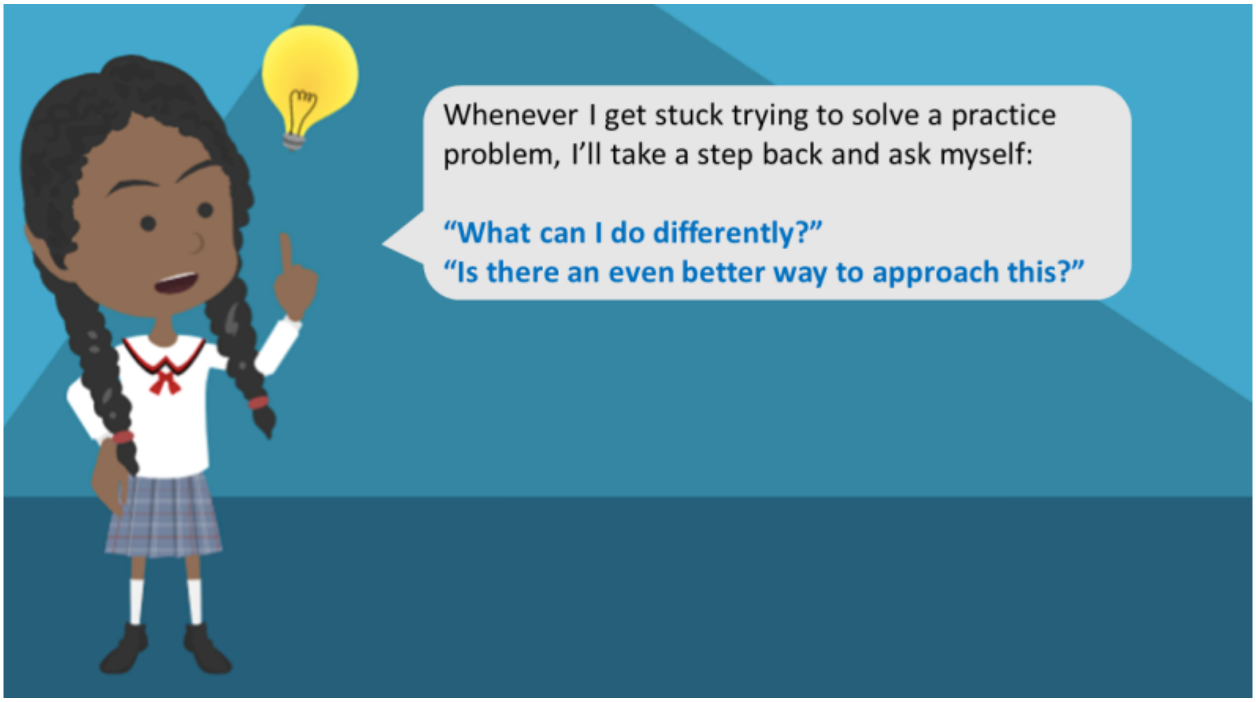


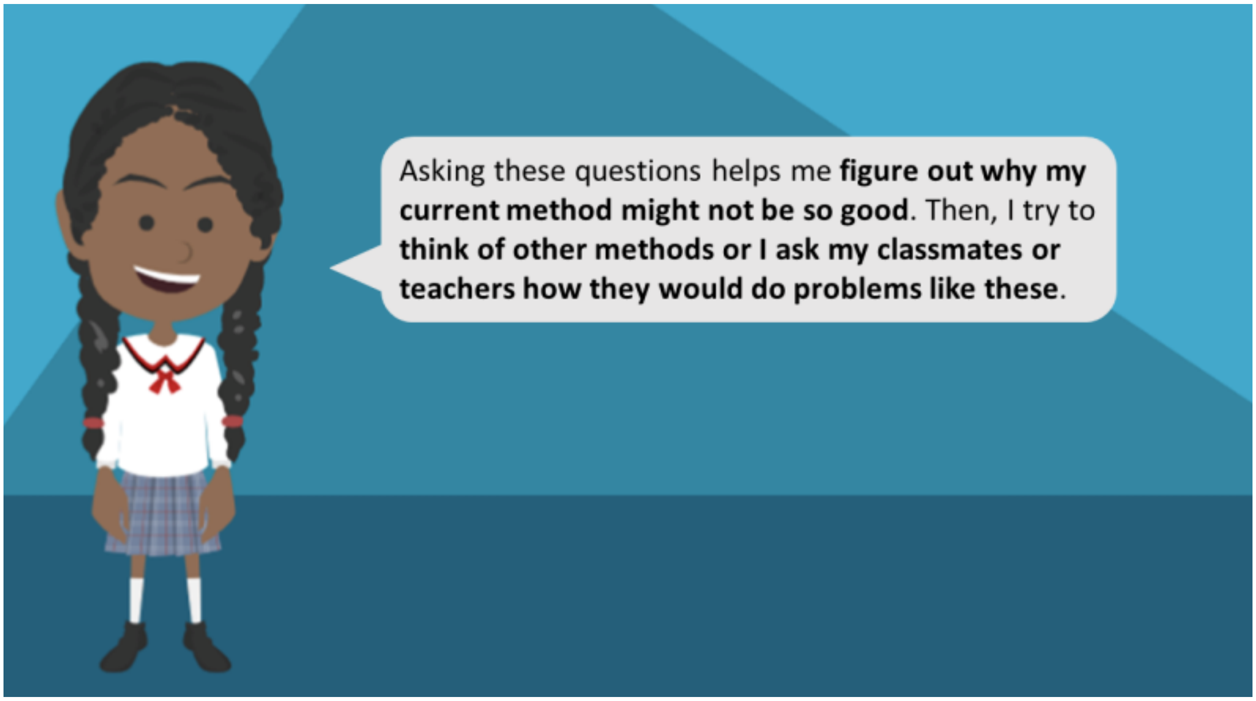

We summarized for students the mindset behind effective learning, and the kinds of questions that they could habitually ask and answer for themselves to learn smarter.

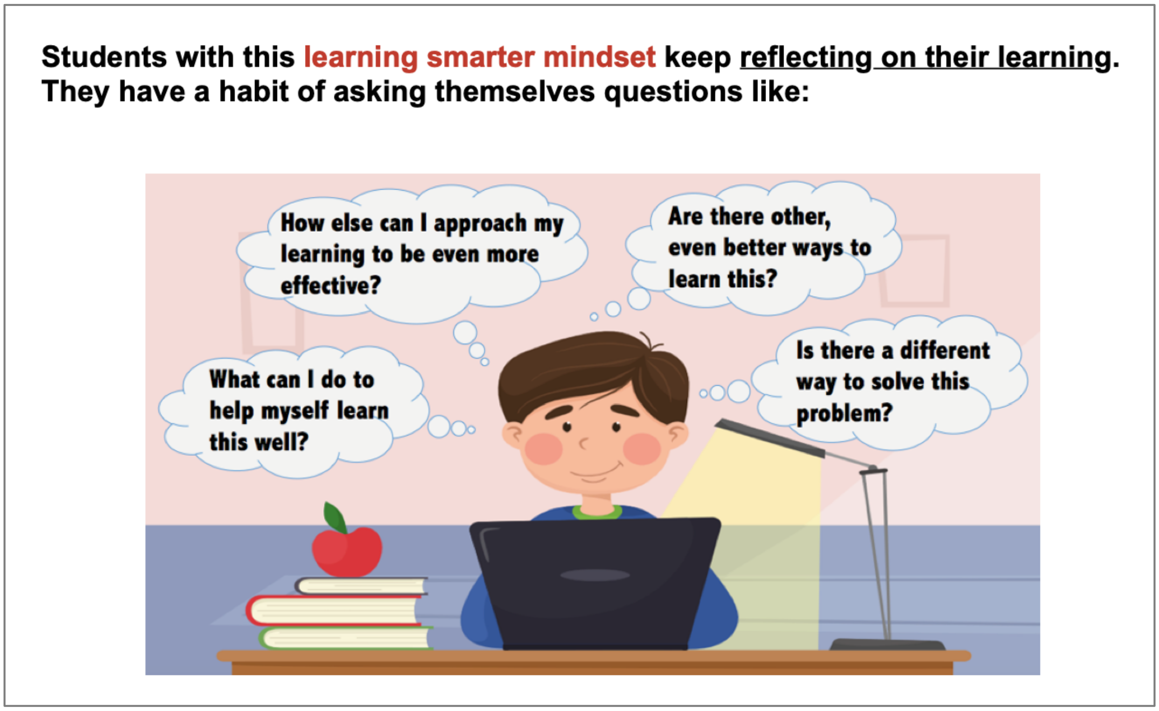


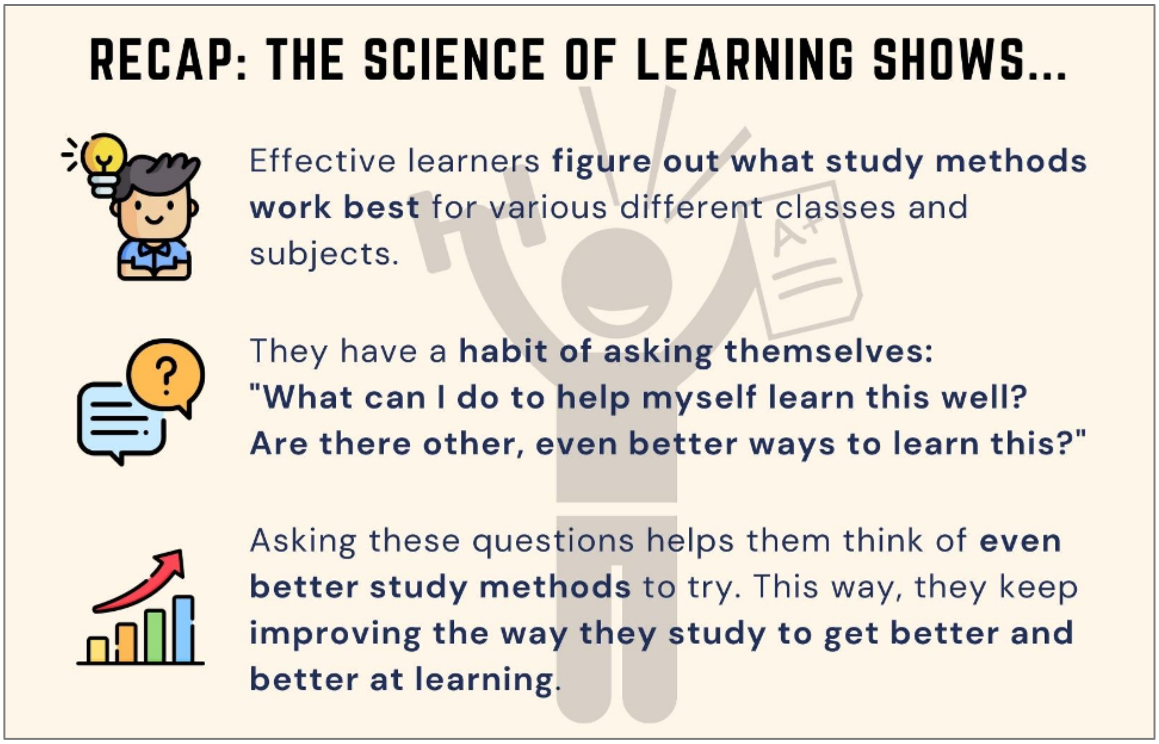

Supplement: Supplementary file 1 — Supplementary Information [file 41539_2025_367_MOESM1_ESM.pdf]
